# Tankyrase represses autoinflammation through the attenuation of TLR2 signaling

**DOI:** 10.1172/JCI140869

**Published:** 2022-04-01

**Authors:** Yoshinori Matsumoto, Ioannis D. Dimitriou, Jose La Rose, Melissa Lim, Susan Camilleri, Napoleon Law, Hibret A. Adissu, Jiefei Tong, Michael F. Moran, Andrzej Chruscinski, Fang He, Yosuke Asano, Takayuki Katsuyama, Ken-ei Sada, Jun Wada, Robert Rottapel

**Affiliations:** 1 Princess Margaret Cancer Centre, University Health Network, University of Toronto, Toronto, Ontario, Canada.; 2 Department of Nephrology, Rheumatology, Endocrinology and Metabolism, Okayama University Graduate School of Medicine, Dentistry and Pharmaceutical Sciences, Okayama, Japan.; 3 Centre for Modeling Human Disease, Toronto Centre for Phenogenomics, Toronto, Ontario, Canada.; 4 Labcorp Early Development Laboratories Inc., Chantilly, Virginia, USA.; 5 Program in Cell Biology, The Hospital for Sick Children, Department of Molecular Genetics,; 6 Multi-Organ Transplant Program, University Health Network,; 7 Department of Medicine,; 8 Department of Medical Biophysics, and; 9 Department of Immunology, University of Toronto, Toronto, Ontario, Canada.; 10 Division of Rheumatology, St. Michael’s Hospital, Toronto, Ontario, Canada.

**Keywords:** Autoimmunity, Inflammation, Cytokines

## Abstract

Dysregulation of Toll-like receptor (TLR) signaling contributes to the pathogenesis of autoimmune diseases. Here, we provide genetic evidence that tankyrase, a member of the poly(ADP-ribose) polymerase (PARP) family, negatively regulates TLR2 signaling. We show that mice lacking tankyrase in myeloid cells developed severe systemic inflammation with high serum inflammatory cytokine levels. We provide mechanistic evidence that tankyrase deficiency resulted in tyrosine phosphorylation and activation of TLR2 and show that phosphorylation of tyrosine 647 within the TIR domain by SRC and SYK kinases was critical for TLR2 stabilization and signaling. Last, we show that the elevated cytokine production and inflammation observed in mice lacking tankyrase in myeloid cells were dependent on the adaptor protein 3BP2, which is required for SRC and SYK activation. These data demonstrate that tankyrase provides a checkpoint on the TLR-mediated innate immune response.

## Introduction

The Toll-like receptors (TLRs) recognize pathogen-associated molecular patterns (PAMPs) derived from microbes to drive the innate immune response (1–3). TLR activation induces NF-κB, a master transcription factor that controls cytokine production, adaptive immune response, tissue repair, and maintenance of mucosal and commensal homeostasis (4–6). Dysregulated TLR signaling has been associated with models of rheumatoid arthritis, systemic lupus erythematosus, and inflammatory bowel disease (6–13).

Tankyrase (tankyrase 1) and its paralog tankyrase 2 are members of the poly(ADP-ribose) polymerase (PARP) family, which catalyzes the addition of ADP-ribose from NAD^+^ to its substrates. Tankyrase-mediated ADP-ribosylation of substrates such as AXIN, AMOT, SH3BP5, and 3BP2 creates a recognition site for the E3 ubiquitin ligase RNF146, leading to substrate ubiquitylation and subsequent proteasomal degradation (14–17).

Gain-of-function missense mutations in the *SH3BP2* gene are associated with cherubism, a rare hereditary syndrome characterized by severe craniofacial developmental defects in children (15, 18). Cherubism mutations uncouple the adaptor protein 3BP2 from tankyrase binding and RNF146-mediated ubiquitylation, resulting in 3BP2 protein stabilization. 3BP2 binds to and activates SRC family kinases through engagement of the SH3 domain. Cherubism mutations lead to the hyperactivation of the 3BP2-associated signaling proteins SRC, SYK, and VAV (15, 18, 19) and a gain-of-function phenotype of macrophages and osteoclasts. Cherubism mice are more responsive to PAMP-induced cytokine production (20–22), demonstrating that 3BP2 potentiates TLR signaling.

In the present study, we show that 3BP2 is a component of the TLR2 signaling complex. 3BP2 recruits SRC and SYK kinases to TLR2 and is required for tyrosine 647 phosphorylation within the TIR domain. Phosphorylation of tyrosine 647 stabilizes TLR2 protein expression by the suppression of TLR2 ubiquitylation and enhances the interaction of TLR2 with MyD88. We show that tyrosine 647 phosphorylation is required for optimum activation of NF-κB. Mice lacking tankyrase in myeloid cells develop severe inflammatory bowel disease and elevated serum cytokine levels. Tankyrase-deficient macrophages exhibit enhanced TLR activation and inflammatory cytokine production. Loss of tankyrase results in SRC- and SYK-mediated tyrosine phosphorylation of TLR2. Last, we show that the inflammatory phenotype observed in tankyrase-deficient mice in myeloid cells is rescued by deletion of 3BP2, which is required for SRC and SYK activation. These data show that 3BP2, SRC, and SYK are components of the proximal signaling machinery associated with TLR2 and are negatively regulated by tankyrase. These data uncover a pivotal gating function of tankyrase in TLR2-mediated innate immunity and autoinflammation.

## Results

### TIR domain tyrosines are required for optimal TLR2 signaling.

**Figure 1:**
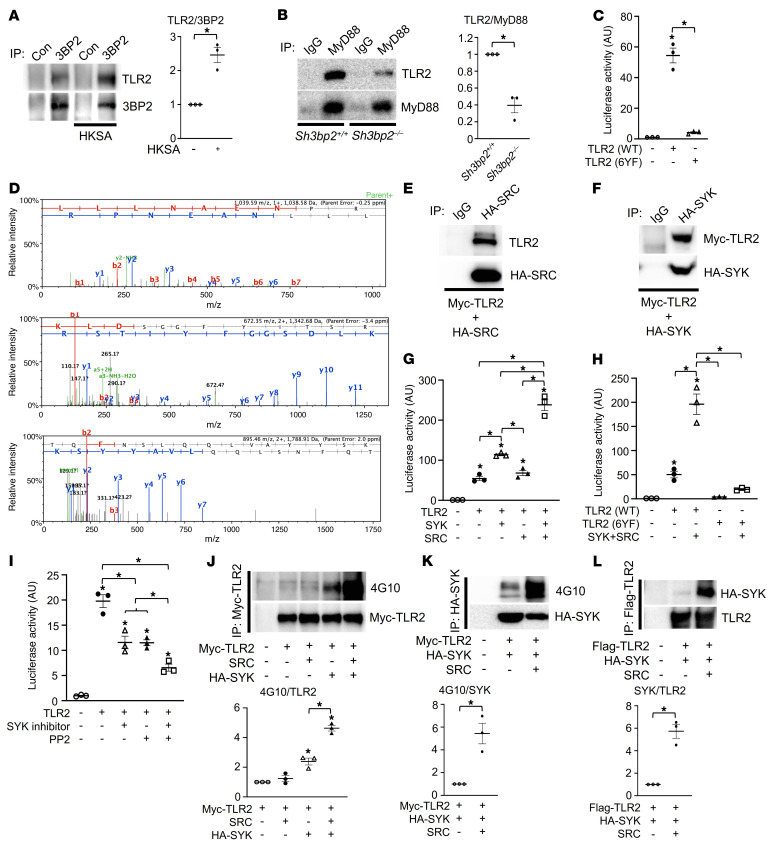
TIR domain tyrosines are required for optimal TLR2 signaling. (**A**) Primary murine macrophages derived from WT mice were starved for 12 hours with 0.1% FBS and cultured in the presence or absence of HKSA (10^7^ cells/mL) for 20 minutes, and 3BP2 immune complexes were probed with the indicated antibodies for Western blot analysis. (**B**) Primary murine macrophages derived from *Sh3bp2^+/+^* and *Sh3bp2^–/–^* mice were starved for 12 hours with 0.1% FBS and cultured in the presence of HKSA (10^7^ cells/mL) for 20 minutes, and MyD88 immune complexes were probed with the indicated antibodies for Western blot analysis. (**C**) Luciferase activity from an NF-κB reporter assay in HEK293T cells cotransfected with the indicated constructs; *n =* 3. (**D**) Three tandem mass spectra of SRC-unique peptides in a TLR2 AP-MS sample. The y and b series of ions are indicated. The sequence of the peptide derived from spectra is shown at the top of each panel. (**E** and **F**) HEK293T cells were cotransfected with the indicated constructs, and HA-SRC (**E**) or HA-SYK (**F**) immune complexes were probed with the indicated antibodies for Western blot analysis. (**G** and **H**) Luciferase activity from an NF-κB reporter assay in HEK293T cells cotransfected with the indicated constructs; *n =* 3. (**I**) Luciferase activity from an NF-κB reporter assay in HEK293T cells cotransfected with the indicated constructs and cultured in the presence or absence of SYK inhibitor (10 μM) and PP2 (10 μM); *n =* 3. (**J**–**L**) HEK293T cells were cotransfected with the indicated constructs, and Myc-TLR2 (**J**), HA-SYK (**K**), or Flag-TLR2 (**L**) immune complexes were probed with the indicated antibodies for Western blot analysis. *P* values were determined by unpaired *t* test (**A**, **B**, **K**, and **L**) or ANOVA with Tukey-Kramer post hoc test (**C** and **G**–**J**). Data are presented as mean ± SEM. *P < 0.05.

Enhanced PAMP-mediated cytokine production observed in macrophages derived from cherubism mice together with diminished cytokine production in 3BP2-deficient mice suggested a role for 3BP2 in TLR signaling (20–22). Since macrophages derived from cherubism mice are particularly sensitive to ligands for TLR2 and TLR4 (21), we investigated whether 3BP2 could interact with TLR2 and the TLR2-associated signaling proteins. We first confirmed that the TLR2 signaling pathway is induced through formation of the TLR2:MyD88 complex by its ligand HKSA in primary murine macrophages as reported previously (refs. 23, 24, and Supplemental Figure 1A; supplemental material available online with this article; https://doi.org/10.1172/JCI140869DS1). Notably, we found that 3BP2 coimmunoprecipitated with TLR2 that was further induced by HKSA in primary murine macrophages (Figure 1A). We demonstrated the binary interaction between 3BP2 and TLR2, MyD88, and TRAF6 in the HEK293T overexpression system (Supplemental Figure 1, B–D). Deletion of 3BP2 in primary murine macrophages disrupted the interaction between TLR2 and MyD88 mediated by PAMPs (Figure 1B), suggesting that 3BP2 is a component of the TLR2 signaling complex. Further, these data show that 3BP2 is required for the ligand-induced formation of the fully mature TLR2 signaling complex.

The Toll/IL-1 receptor (TIR) domain is a region of common homology between the IL-1 receptor and TLR family members (25, 26). The TIR domain of TLR2 is a cytoplasmic module required for signal transduction and subsequent activation of NF-κB (27–29). Among 6 tyrosines in the cytoplasmic domain of TLR2, the TIR domain contains 5 tyrosines, 4 of which are highly conserved across species and other TIR domain–containing proteins including TLR4 and TLR7, suggesting that they may have conserved function in TLR signaling. We mutated all 6 cytoplasmic tyrosine residues to phenylalanine (6YF) and expressed this mutant in HEK293T cells to determine whether these residues are necessary for NF-κB activation. Ectopic expression of wild-type TLR2 (WT) in HEK293T cells induced NF-κB transcriptional activity, while the TLR2 (6YF) mutant was transcriptionally inactive (Figure 1C and Supplemental Figure 1E), demonstrating a critical role for tyrosine residues in mediating TLR2 signaling. To identify TLR2-interacting proteins that may depend on one or more of the cytoplasmic tyrosines, we performed mass spectrometry to detect proteins that differentially interacted with TLR2 (WT) compared with the TLR2 (6YF) mutant. We identified the 3BP2-associated protein SRC tyrosine kinase as a binding partner for TLR2 (WT) but not for the TLR2 (6YF) mutant (Figure 1D, Supplemental Figure 1F, and Supplemental Table 1). We then validated whether the 3BP2-associated proteins SRC and SYK were components of the TLR2 signaling complex and observed that SRC and SYK coprecipitated with TLR2 (Figure 1, E and F).

We next examined the dependency of TLR2-induced NF-κB transcriptional activity on the expression of SRC and SYK. SYK expression doubled the level of TLR2-induced NF-κB transcriptional activity, while the combination of both SYK and constitutively active c-SRC (Y529F) expression increased TLR2-dependent NF-κB activity 5-fold (Figure 1G). The increase in TLR2-dependent NF-κB activity by SYK and SRC was lost in the TLR2 (6YF) mutant (Figure 1H). Further, pharmacologic inhibition of SYK and SRC diminished NF-κB activity (Figure 1I and Supplemental Figure 1G). These data suggest that the TIR domain tyrosine residues may be modified by SRC and SYK to optimize TLR2 signaling. To determine whether TLR2 is directly phosphorylated, we examined the tyrosine phosphorylation levels of TLR2 in the presence of ectopically expressed SRC and SYK. TLR2 was phosphorylated by SYK, which was further enhanced by coexpression of a constitutively active mutant form of SRC (Figure 1J and Supplemental Figure 1H). SRC enhanced SYK phosphorylation (Figure 1K and Supplemental Figure 1I) and increased the interaction between SYK and TLR2 (Figure 1L and Supplemental Figure 1J) in an ectopic expression system. Together these data demonstrate that TLR2 is tyrosine phosphorylated in an SRC- and SYK-dependent manner and that the cytoplasmic tyrosine residues are critical for optimal NF-κB activation.

### TIR domain tyrosines are required for the stability of TLR2.

**Figure 2:**
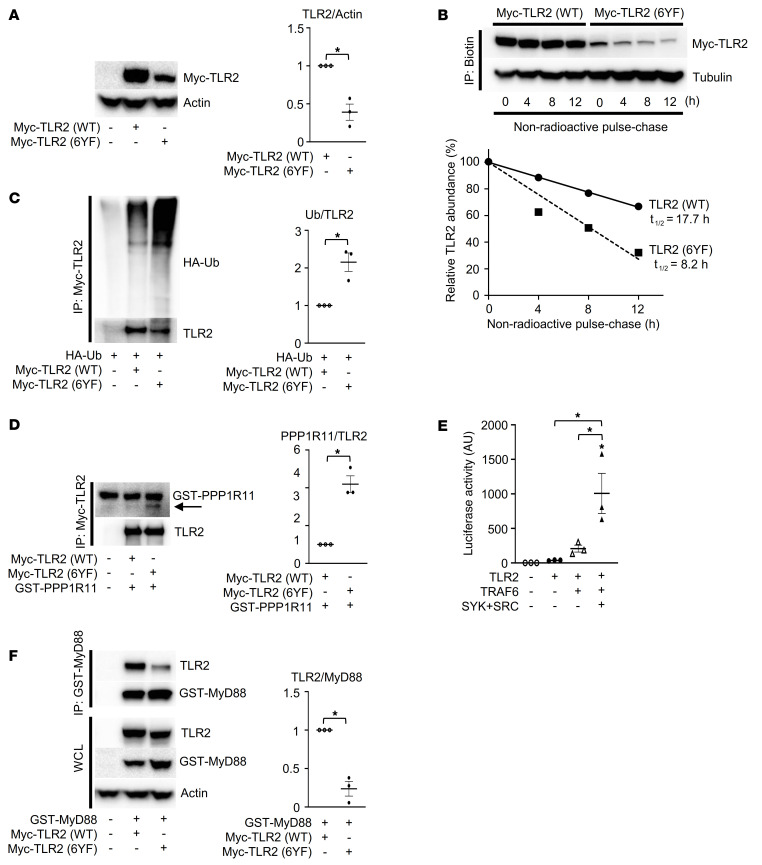
TIR domain tyrosines are required for the stability of TLR2. (**A**) Whole-cell lysates from HEK293T cells cotransfected with the indicated constructs were probed with the indicated antibodies for Western blot analysis. (**B**) Nonradioactive pulse-chase assay to analyze turnover of TLR2 (WT) or TLR2 (6YF) protein labeled with Click-IT Metabolic Labeling. Biotinylated proteins were probed with the indicated antibodies, and the percentages of TLR2 protein levels were plotted as a function of time. (**C**, **D**, and **F**) HEK293T cells were cotransfected with the indicated constructs, and Myc-TLR2 (**C** and **D**) or GST-MyD88 (**F**) immune complexes and whole-cell lysates (WCL) were probed with the indicated antibodies for Western blot analysis. (**E**) Luciferase activity from an NF-κB reporter assay in HEK293T cells cotransfected with the indicated constructs; *n =* 3. *P* values were determined by unpaired *t* test (**A**, **C**, **D**, and **F**) or ANOVA with Tukey-Kramer post hoc test (**E**). Data are presented as mean ± SEM. *P < 0.05.

We noted that the protein expression of the TLR2 (6YF) mutant was significantly reduced (Figure 2A). To determine whether the tyrosine phosphorylation of TLR altered the stability of TLR2, we performed a pulse-chase assay in TLR2 (WT)– or TLR2 (6YF)–expressing 293T cells. As shown in Figure 2B, the half-life of TLR2 (WT) protein was 17.7 hours compared with 8.2 hours for the 6YF mutant form of the protein that was similarly observed in a cycloheximide-chase experiment (Supplemental Figure 2A). We examined how cytoplasmic tyrosines control TLR2 protein turnover through ubiquitylation. We noted that the level of TLR2 ubiquitylation of the TLR2 (6YF) mutant was elevated compared with that of the WT form of the receptor (Figure 2C), suggesting that TLR2 expression may be regulated by ubiquitylation in a tyrosine-dependent manner. The E3 ubiquitin ligase PPP1R11 has recently been reported to control the protein expression levels of TLR2 through ubiquitylation (30). Consistent with the ubiquitylation levels of TLR2 in Figure 2C, we observed an increased interaction between PPP1R11 and TLR2 (6YF) compared with TLR2 (WT) (Figure 2D and Supplemental Figure 2B), demonstrating that cytoplasmic tyrosines are required for stabilization of TLR2 in part through the suppression of ubiquitin-mediated proteolysis.

We next investigated whether SRC and SYK kinases potentiate TLR2 signaling. The combined expression of SRC and SYK enhanced TRAF6-mediated NF-κB stimulation 4-fold (Figure 2E), showing that SRC, SYK, MyD88, and TRAF6 comprise the downstream signaling machinery for TLR2 required for its optimum activation of NF-κB. Mutation of all the cytoplasmic domain tyrosines or combined use of SRC and SYK inhibitors reduced the interaction of TLR2 with MyD88 (Figure 2F and Supplemental Figure 2C). These data show that cytoplasmic tyrosines are required for TLR2 stabilization and the assembly of the TLR2 signaling complex.

### TLR2 tyrosine 647 is a phospho-switch that regulates TLR2 stabilization and activation.

**Figure 3:**
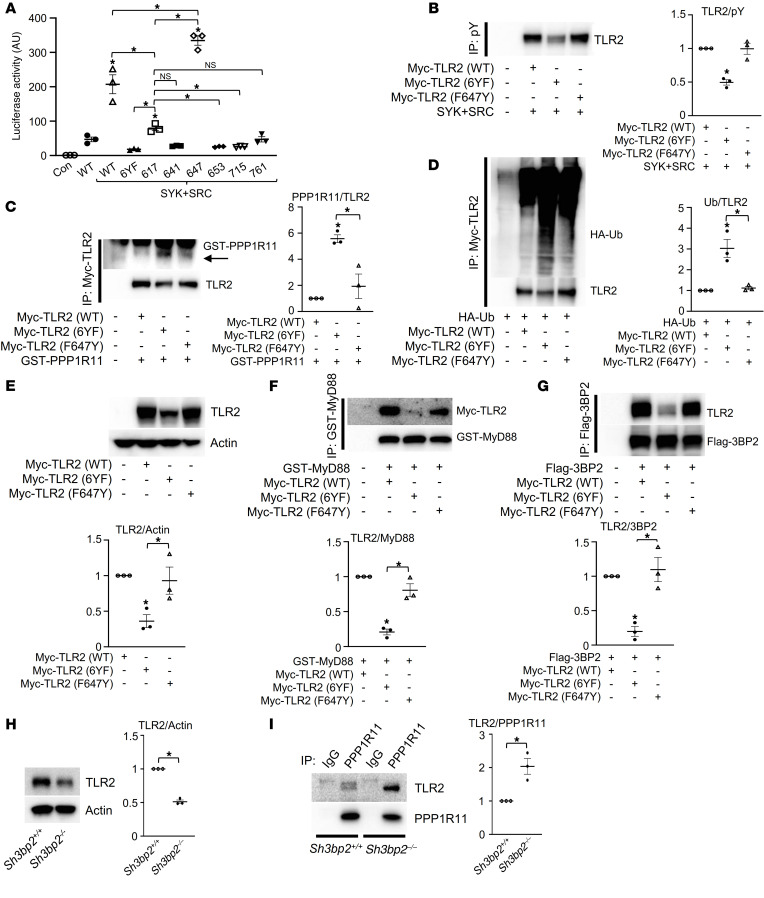
TLR2 tyrosine 647 is a phospho-switch that regulates TLR2 stabilization and activation. (**A**) Luciferase activity from an NF-κB reporter assay in HEK293T cells cotransfected with the indicated constructs; *n =* 3. (**B**) HEK293T cells cotransfected with the indicated constructs were treated with 10 μM MG132 for 8 hours before collection of cell lysates. pY immune complexes were probed with an anti-TLR2 antibody. (**C**, **F**, and **G**) HEK293T cells were cotransfected with the indicated constructs, and Myc-TLR2 (**C**), GST-MyD88 (**F**), or Flag-3BP2 (**G**) immune complexes were probed with the indicated antibodies for Western blot analysis. (**D**) HEK293T cells cotransfected with the indicated constructs were treated with 10 μM MG132 for 4 hours before collection of cell lysates. Myc-TLR2 immune complexes were probed with an anti-HA antibody. (**E**) Whole-cell lysates from HEK293T cells cotransfected with the indicated constructs were probed with the indicated antibodies for Western blot analysis. (**H**) Whole-cell lysates from primary murine macrophages derived from *Sh3bp2^+/+^* and *Sh3bp2^–/–^* mice were probed with the indicated antibodies for Western blot analysis. (**I**) Primary murine macrophages derived from *Sh3bp2^+/+^* and *Sh3bp2^–/–^* mice were lysed, and PPP1R11 immune complexes were probed with the indicated antibodies for Western blot analysis. *P* values were determined by unpaired *t* test (**H** and **I**) or ANOVA with Tukey-Kramer post hoc test (**A**–**G**). Data are presented as mean ± SEM. *P < 0.05.

We investigated the molecular mechanism by which SRC- and SYK-mediated phosphorylation of tyrosine residues within the TLR2 cytoplasmic domain promotes TLR2 activation. We first created tyrosine add-back variants of the TLR2 (6YF) mutant to determine which tyrosine(s) were sufficient to mediate TLR2 activation of NF-κB. We found that Y647 in the TIR domain was sufficient to fully restore NF-κB activation (Figure 3A) as well as TLR2 phosphorylation levels (Figure 3B and Supplemental Figure 3A). This observation suggests that most of the tyrosine phosphorylation in the TIR domain can be attributed to Y647. The Y647 add-back on the TLR2 (6YF) background was sufficient to decrease the interaction with PPP1R11 to a level similar to that observed with TLR2 (WT) (Figure 3C). In support of a functional interaction between TLR2 and PPP1R11, Y647 was also sufficient to restore TLR2 ubiquitylation and total protein expression to WT levels (Figure 3, D and E). Additionally, we observed that the interaction of MyD88 with TLR2, reduced in the 6YF mutant in Figure 2F, was restored to WT levels by the add-back of Y647 (Figure 3F and Supplemental Figure 3B).

Last, we examined the role of tyrosine 647 in recruiting the adaptor protein 3BP2 to TLR2. The interaction of 3BP2 with TLR2 was disrupted in the TLR2 (6YF) mutant but restored in the Y647 add-back variant (Figure 3G and Supplemental Figure 3C). Moreover, we observed that the steady-state level of TLR2 was reduced in primary murine macrophages lacking 3BP2 through the enhancement of the interaction with PPP1R11 (Figure 3, H and I). These data suggest that Y647 phosphorylation in the TIR domain by SRC and SYK serves to regulate the mutual exclusive interaction of TLR2 with the ubiquitin ligase PPP1R11 and MyD88 and that the tyrosine-mediated signaling complex of TLR2 is dependent on 3BP2.

### Mice lacking tankyrase in myeloid cells exhibit systemic inflammation.

**Figure 4:**
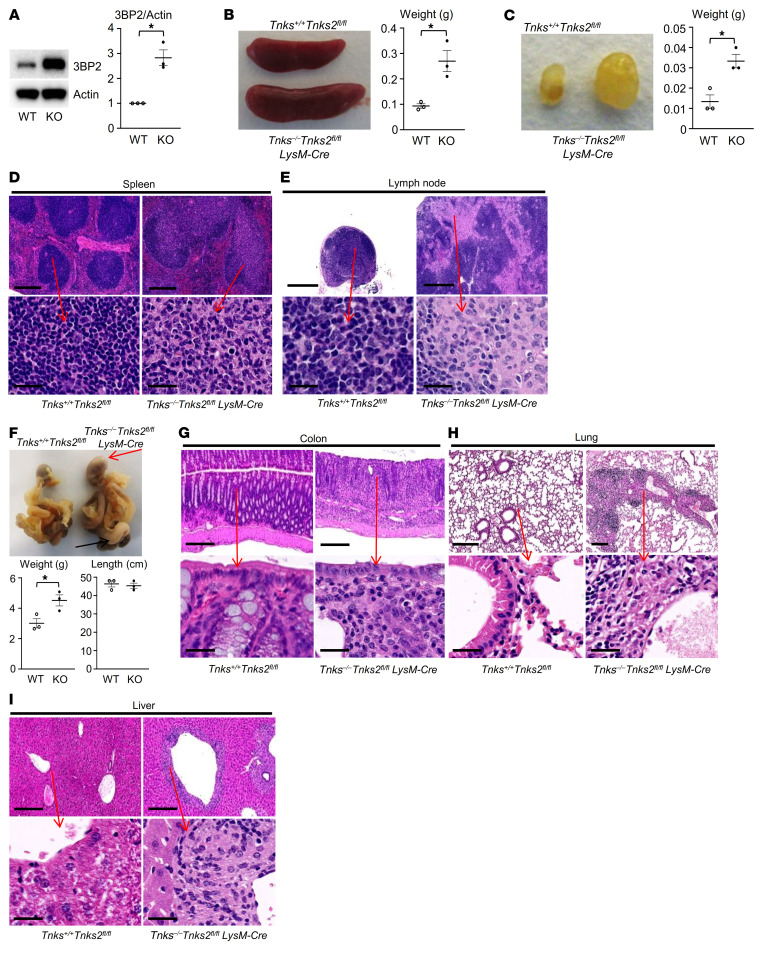
Mice lacking tankyrase in myeloid cells exhibit systemic inflammation. (**A**) Whole-cell lysates from primary murine macrophages derived from *Tnks^+/+^* *Tnks2^fl/fl^* (WT) and *Tnks^–/–^* *Tnks2^fl/fl^ LysM-Cre* (KO) mice were probed with the indicated antibodies for Western blot analysis. (**B**, **C**, and **F**) Representative images of spleen (**B**), lymph node (**C**), and gut (**F**) from 12-week-old *Tnks^+/+^* *Tnks2^fl/fl^* and *Tnks^–/–^* *Tnks2^fl/fl^ LysM-Cre* mice. The weight of these organs and the length of the gut and are shown with the statistical analysis. Red arrow indicates the stomach, and black arrow indicates the colon. (**D**, **E**, and **G**–**I**) Lower-magnification (top) and higher-magnification (bottom) images of H&E staining of spleen (**D**), lymph node (**E**), colon (**G**), lung (**H**), and liver (**I**) from 12-week-old *Tnks^+/+^* *Tnks2^fl/fl^* and *Tnks^–/–^* *Tnks2^fl/fl^ LysM-Cre* mice. Scale bars, top: 250 μm (**D** and **G**–**I**), 500 μm (**E**); scale bars, bottom: 25 μm. *P* values were determined by unpaired *t* test. Data are presented as mean ± SEM. *P < 0.05.

We previously reported that the steady-state levels of 3BP2 are negatively regulated by tankyrase-mediated ADP-ribosylation and subsequent ubiquitylation by RNF146 (15). The role of tankyrase in adult tissues has been difficult to ascertain since a double knockout of tankyrase 1 (*Tnks*) and tankyrase 2 (*Tnks2*) in mice is embryonic lethal while knockout of either *Tnks* or *Tnks2* shows only mild phenotypes (31–35), demonstrating that the 2 forms of tankyrase share some functional redundancy. To investigate the function of tankyrase within the myeloid monocytic lineage, we generated conditional double knockout (KO) mice (*Tnks^–/–^* *Tnks2^fl/fl^ LysM-Cre*) in which endogenous tankyrase 1 was excised in all tissues including germ cells while tankyrase 2 was deleted in the myeloid monocytic lineage (Supplemental Figure 4, A–C). We found that the expression levels of 3BP2 protein were elevated in *Tnks^–/–^* *Tnks2^fl/fl^ LysM-Cre* macrophages compared with *Tnks^+/+^* *Tnks2^fl/fl^* (WT) cells (Figure 4A). *Tnks^–/–^* *Tnks2^fl/fl^ LysM-Cre* mice were smaller, failed to thrive, suffered from diarrhea, and developed swollen eyelids indicative of blepharitis. *Tnks^–/–^* *Tnks2^fl/fl^ LysM-Cre* mice exhibited splenomegaly (Figure 4B) and lymphadenopathy (Figure 4C). Infiltration of mononuclear histiocytes with pale cytoplasm resulted in loss of the mantle zone and expansion of the splenic white pulp as well as the sinuses within the lymph nodes, and the splenic red pulp was expanded by extramedullary hematopoiesis (Figure 4, D and E). The stomach and intestines from *Tnks^–/–^* *Tnks2^fl/fl^ LysM-Cre* mice were grossly thickened and discolored, and microscopically, the lamina propria was expanded by mononuclear inflammatory cells replacing and effacing glandular tissue and villi (Figure 4, F and G, and Supplemental Figure 4, D–F). Perivascular mononuclear infiltrates and lymphocyte clusters were observed in lung and liver from *Tnks^–/–^* *Tnks2^fl/fl^ LysM-Cre* mice (Figure 4, H and I).

### Tankyrase regulates inflammation by controlling innate and adaptive immunity.

**Figure 5:**
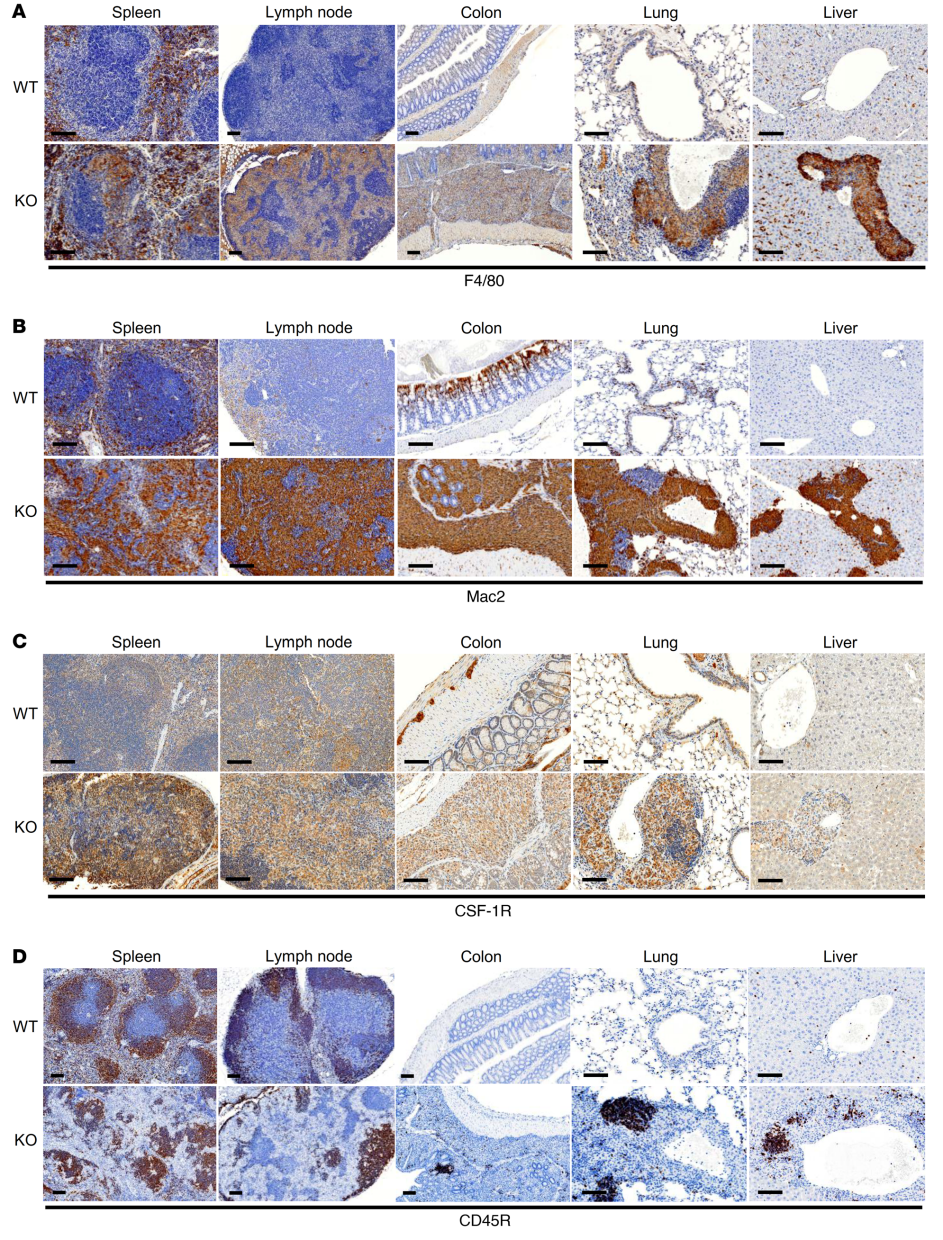
Tankyrase regulates inflammation by controlling innate and adaptive immunity. (**A**–**D**) F4/80 (**A**), Mac2 (**B**), CSF-1R (**C**), and CD45R (**D**) immunostaining of spleen, lymph node, colon, lung, and liver from 12-week-old *Tnks^+/+^* *Tnks2^fl/fl^* (WT) and *Tnks^–/–^* *Tnks2^fl/fl^ LysM-Cre* (KO) mice. Scale bars: 100 μm.

**Figure 6:**
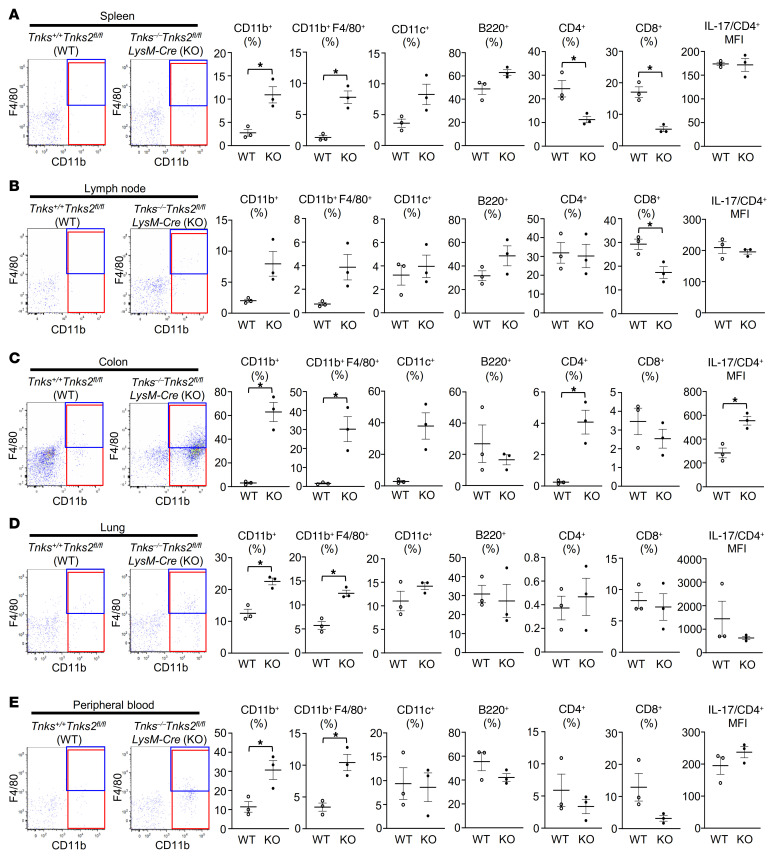
Tankyrase regulates inflammation by controlling innate and adaptive immunity. (**A**–**E**) Flow cytometric analysis of CD11b, F4/80, CD11c, B220, CD4, and CD8 expression on the cell surface and IL-17 expression in CD4-positive cells of spleen (**A**), lymph node (**B**), colon (**C**), lung (**D**), and peripheral blood (**E**) from 12-week-old *Tnks^+/+^* *Tnks2^fl/fl^* (WT) and *Tnks^–/–^* *Tnks2^fl/fl^ LysM-Cre* (KO) mice; *n =* 3. Representative flow cytometry plots of CD11b- and/or F4/80-expressing cells (left), the frequency (middle, percent cells), and the mean fluorescence intensity (MFI) of IL-17 (far right) are presented as mean ± SEM. *P* values were determined by unpaired *t* test. *P < 0.05.

To define the infiltrating immune cells in the visceral organs in *Tnks^–/–^* *Tnks2^fl/fl^ LysM-Cre* mice, we performed immunohistochemistry (IHC) staining of F4/80 (macrophage), Mac2 (macrophage), CSF-1R (monocyte), CD45R (B lymphocyte), myeloperoxidase (MPO; neutrophil), and eosinophil peroxidase (EPX; eosinophil) of spleen, lymph node, colon, lung, and liver from 12-week-old *Tnks^+/+^* *Tnks2^fl/fl^* or *Tnks^–/–^* *Tnks2^fl/fl^ LysM-Cre* mice. We observed that F4/80- and Mac2- as well as CSF-1R–positive activated monocytes/macrophages predominantly infiltrated these organs in *Tnks^–/–^* *Tnks2^fl/fl^ LysM-Cre* mice, leading to destruction of lymphoid follicles in spleen and lymph node and reactive recruitment of CD45R-positive B lymphocytes in the enlarged colon and the perivascular regions of lung and liver (Figure 5, A–D). In distinction, infiltration of MPO-positive neutrophils and EPX-positive eosinophils was comparable in *Tnks^–/–^* *Tnks2^fl/fl^ LysM-Cre* and *Tnks^+/+^* *Tnks2^fl/fl^* mice (Supplemental Figure 5, A and B). Consistent with these IHC data, flow cytometric analysis of spleen, lymph node, colon, lung, and peripheral blood from *Tnks^–/–^* *Tnks2^fl/fl^ LysM-Cre* mice showed increased frequency and absolute cell numbers of CD11b- and F4/80-positive cells infiltrating these organs (Figure 6, A–E, and Supplemental Figure 6, A–D). The number of B220-positive B lymphocytes was increased in spleen and colon while the mean fluorescence intensity of IL-17 but not IL-6 and TNF-α (data not shown) in CD4-positive T lymphocytes was increased only in the colon from *Tnks^–/–^* *Tnks2^fl/fl^ LysM-Cre* mice compared with *Tnks^+/+^* *Tnks2^fl/fl^* mice (Figure 6, A–E, and Supplemental Figure 6, A–G). These data demonstrate that deletion of tankyrase in myeloid cells results in the expansion of Mac2-, CSF-1R–, CD11b-, and F4/80-positive myeloid monocytic cells and the reactive recruitment of T and B lymphocytes into the gut and visceral organs.

### Tankyrase restrains TLR signaling and the production of inflammatory cytokines.

**Figure 7:**
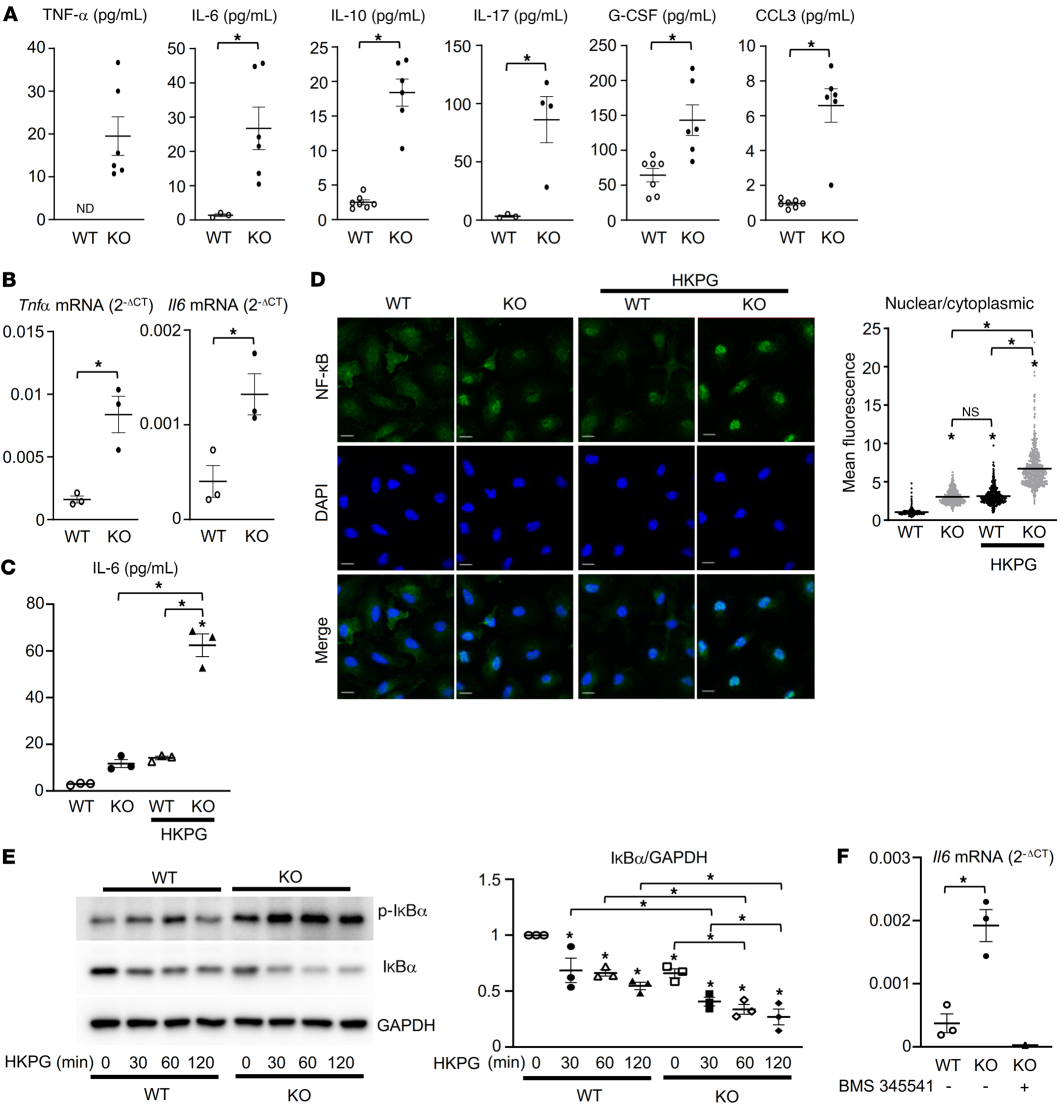
Tankyrase restrains TLR signaling and production of inflammatory cytokines. (**A**) Serum levels of TNF-α, IL-6, IL-10, IL-17, G-CSF, and CCL3 in 12-week-old *Tnks^+/+^* *Tnks2^fl/fl^* (WT) and *Tnks^–/–^* *Tnks2^fl/fl^ LysM-Cre* (KO) mice. ND, not detected. (**B**) Quantitative real-time PCR (qPCR) analysis of *Tnfa* and *Il6* mRNA expression in primary murine macrophages derived from *Tnks^+/+^* *Tnks2^fl/fl^* (WT) and *Tnks^–/–^* *Tnks2^fl/fl^ LysM-Cre* (KO) mice; *n =* 3. (**C**) Primary murine macrophages derived from *Tnks^+/+^Tnks2^fl/fl^* (WT) and *Tnks^–/–^Tnks2^fl/fl^ LysM-Cre* (KO) mice were starved for 12 hours with 0.1% FBS and cultured in the presence or absence of HKPG (10^7^ cells/mL) for 24 hours. IL-6 protein levels in the culture supernatant were measured by ELISA. *n =* 3. (**D**) Primary murine macrophages derived from *Tnks^+/+^* *Tnks2^fl/fl^* (WT) and *Tnks^–/–^* *Tnks2^fl/fl^ LysM-Cre* (KO) mice, starved for 12 hours with 0.1% FBS and cultured in the presence or absence of HKPG (10^7^ cells/mL) for 2 hours, were stained by immunofluorescence. Scale bars: 10 μm. Images of intracellular NF-κB (green) and nuclei (blue) are representative of 3 independent experiments. Quantification of NF-κB cytoplasmic and nuclear expression is presented as the ratio of nuclear/cytoplasmic mean fluorescence values as described previously (61). (**E**) Whole-cell lysates from primary murine macrophages derived from *Tnks^+/+^* *Tnks2^fl/fl^* (WT) and *Tnks^–/–^* *Tnks2^fl/fl^ LysM-Cre* (KO) mice, starved for 12 hours with 0.1% FBS and cultured in the presence of HKPG (10^7^ cells/mL) for 0–120 minutes, were probed with the indicated antibodies for Western blot analysis. (**F**) qPCR analysis of *Il6* mRNA expression in primary murine macrophages derived from *Tnks^+/+^* *Tnks2^fl/fl^* (WT) and *Tnks^–/–^* *Tnks2^fl/fl^ LysM-Cre* (KO) mice and cultured in the presence or absence of the IKK inhibitor BMS 345541 (10 μM); *n =* 3. *P* values were determined by unpaired *t* test (**A** and **B**) or ANOVA with Tukey-Kramer post hoc test (**C**–**F**). Data are presented as mean ± SEM. *P < 0.05.

We next measured serum cytokine levels in the *Tnks^–/–^* *Tnks2^fl/fl^ LysM-Cre* mice. Under basal conditions, TNF-α, IL-6, IL-10, IL-12, IL-17, G-CSF, and CCL3 were elevated in the serum of *Tnks^–/–^* *Tnks2^fl/fl^ LysM-Cre* mice compared with *Tnks^+/+^* *Tnks2^fl/fl^* mice (Figure 7A), whereas basal levels of IL-4, IL-5, IL-9, CCL2, CCL11, and CXCL1 were similar for both *Tnks^+/+^* *Tnks2^fl/fl^* and *Tnks^–/–^* *Tnks2^fl/fl^ LysM-Cre* mice (Supplemental Figure 7A). GM-CSF, IL-1α, IL-1β, IL-13, IFN-γ, and CCL4 were below detectable levels in both *Tnks^+/+^* *Tnks2^fl/fl^* and *Tnks^–/–^* *Tnks2^fl/fl^ LysM-Cre* mice (data not shown).

To investigate the cellular mechanism underlying the dysregulation of cytokine expression in mice lacking tankyrase in myeloid cells, we measured the in vitro production of cytokines in primary bone marrow–derived macrophages isolated from *Tnks^–/–^* *Tnks2^fl/fl^ LysM-Cre* mice and observed elevated transcripts of *Tnfa* and *Il6* and the IL-6 protein level in the culture supernatant under basal conditions as well as cell lysates of macrophages in comparison with *Tnks^+/+^* *Tnks2^fl/fl^* mice (Figure 7B and Supplemental Figure 7B). These data show that tankyrase normally regulates the basal levels of inflammatory cytokines.

We next queried whether the elevation of cytokine transcripts in tankyrase-deficient macrophages was through activation of the TLR2 signaling pathway and observed that *Tnks^–/–^* *Tnks2^fl/fl^ LysM-Cre* macrophages stimulated with the TLR2 ligand HKPG induced the elevated IL-6 level in the culture supernatant as well as the *Il6* transcript level compared with *Tnks^+/+^* *Tnks2^fl/fl^* cells (Figure 7C and Supplemental Figure 7C). Upon activation with the ligands, TLR2 normally interacts with MyD88 and activates the downstream signaling pathway through phosphorylation of IκBα, an inhibitory component of NF-κB, leading to its proteasome-mediated degradation and release of active NF-κB to the nucleus (36). We therefore performed immunofluorescence of NF-κB and observed nuclear accumulation of NF-κB in *Tnks^+/+^* *Tnks2^fl/fl^* macrophages stimulated with HKPG that was further enhanced in *Tnks^–/–^* *Tnks2^fl/fl^ LysM-Cre* macrophages (Figure 7D). Consistently, phosphorylation of IκBα was enhanced by stimulation with HKPG, leading to degradation of IκBα and subsequent phosphorylation and activation of NF-κB in *Tnks^+/+^* *Tnks2^fl/fl^* macrophages that were further enhanced in *Tnks^–/–^* *Tnks2^fl/fl^ LysM-Cre* cells (Figure 7E and Supplemental Figure 7, D and E). To confirm whether loss of tankyrase in macrophages resulted in activation of the TLR/NF-κB signaling pathway and subsequent inflammatory cytokine production, we cultured tankyrase-null macrophages in the presence or absence of the IκB kinase (IKK) inhibitor BMS 345541 (37). Treatment with BMS 345541 abolished enhanced expression of *Il6* transcripts observed in *Tnks^–/–^* *Tnks2^fl/fl^ LysM-Cre* macrophages (Figure 7F). These results indicate that tankyrase is a regulator of TLR2 responsiveness to stimulatory ligands and that high production of inflammatory cytokines observed in tankyrase-null macrophages results in part from enhanced activation of the NF-κB signaling pathway.

To determine whether the immune dysregulation observed in the *Tnks^–/–^* *Tnks2^fl/fl^ LysM-Cre* mice could lead to a loss of humoral tolerance, we probed autoantigen microarrays that displayed 153 autoantigens (38) with the sera from the knockout mice and observed a constellation of autoantibodies that recognize snRNP-A, C1q, La/SS-B, PM/Scl-75, and RNP/Sm in contrast to those from WT mice (Supplemental Figure 7F). The presence of these autoantibodies was predominantly observed in the sera of female mice (Supplemental Figure 7F). The spectrum of these autoantibodies is frequently observed in patients with systemic lupus erythematosus, scleroderma, and mixed connective tissue disease.

These data demonstrate that the main source of inflammatory cytokines observed in *Tnks^–/–^* *Tnks2^fl/fl^ LysM-Cre* mice arises from the macrophage lineage and that tankyrase protects against the selective loss of humoral tolerance.

### Tyrosine phosphorylation of TLR2 regulates NF-κB–mediated cytokine production.

**Figure 8:**
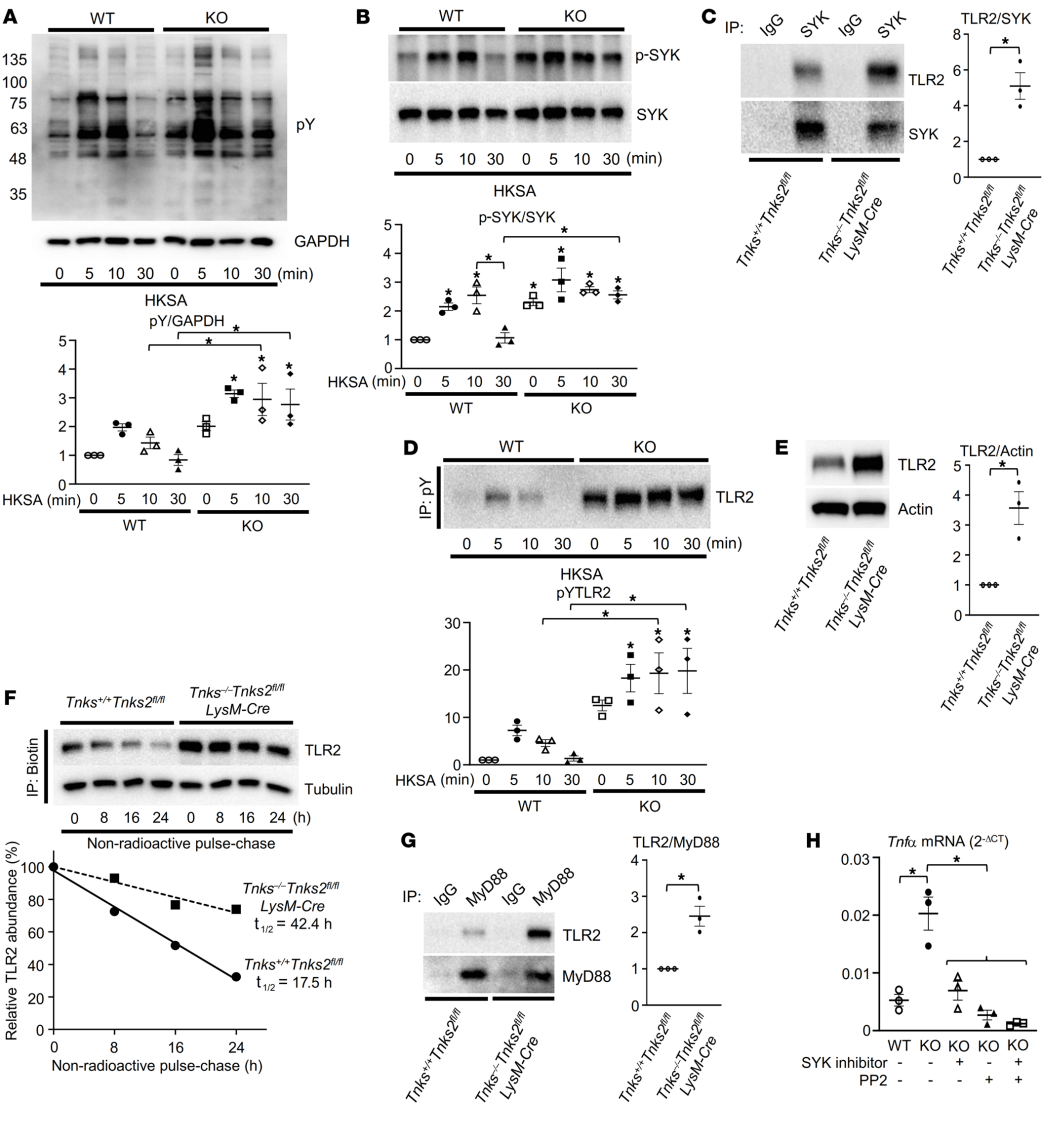
Tyrosine phosphorylation of TLR2 regulates NF-κB–mediated cytokine production. (**A** and **B**) Whole-cell lysates from primary murine macrophages derived from *Tnks^+/+^* *Tnks2^fl/fl^* (WT) and *Tnks^–/–^* *Tnks2^fl/fl^ LysM-Cre* (KO) mice, starved for 12 hours with 0.1% FBS and cultured in the presence of HKSA (10^7^ cells/mL) for 0–30 minutes, were probed with the indicated antibodies for Western blot analysis. (**C** and **G**) Primary murine macrophages derived from *Tnks^+/+^* *Tnks2^fl/fl^* and *Tnks^–/–^* *Tnks2^fl/fl^ LysM-Cre* mice were starved for 12 hours with 0.1% FBS and cultured in the presence of HKSA (10^7^ cells/mL) for 20 minutes, and SYK (**C**) or MyD88 (**G**) immune complexes were probed with the indicated antibodies for Western blot analysis. (**D**) Primary murine macrophages derived from *Tnks^+/+^* *Tnks2^fl/fl^* and *Tnks^–/–^* *Tnks2^fl/fl^ LysM-Cre* mice were starved for 12 hours with 0.1% FBS and cultured in the presence of HKSA (10^7^ cells/mL) for 0–30 minutes, and pY immune complexes were probed with the indicated antibodies for Western blot analysis. (**E**) Whole-cell lysates from primary murine macrophages derived from *Tnks^+/+^* *Tnks2^fl/fl^* and *Tnks^–/–^* *Tnks2^fl/fl^ LysM-Cre* mice were probed with the indicated antibodies for Western blot analysis. (**F**) Nonradioactive pulse-chase assay to analyze turnover of endogenous TLR2 protein labeled with Click-IT Metabolic Labeling in primary murine macrophages derived from *Tnks^+/+^* *Tnks2^fl/fl^* and *Tnks^–/–^* *Tnks2^fl/fl^ LysM-Cre* mice. Biotinylated proteins were probed with the indicated antibodies, and percentages of TLR2 protein levels were plotted as a function of time. (**H**) qPCR analysis of *Tnfa* mRNA expression in primary murine macrophages derived from *Tnks^+/+^* *Tnks2^fl/fl^* (WT) and *Tnks^–/–^* *Tnks2^fl/fl^ LysM-Cre* (KO) mice and cultured in the presence or absence of SYK inhibitor (10–50 μM) and PP2 (10 μM); *n =* 3. *P* values were determined by unpaired *t* test (**C**, **E**, and **G**) or ANOVA with Tukey-Kramer post hoc test (**A**, **B**, **D**, and **H**). Data are presented as mean ± SEM. *P < 0.05.

To investigate the biochemical basis for the autoinflammatory phenotype that we observed in the *Tnks^–/–^* *Tnks2^fl/fl^ LysM-Cre* mice, we stimulated *Tnks^+/+^* *Tnks2^fl/fl^* (WT) and *Tnks^–/–^* *Tnks2^fl/fl^ LysM-Cre* (KO) macrophages with the TLR2 ligand HKSA and monitored total cellular phosphotyrosine levels (Figure 8A). Importantly, we observed that the general level of phosphorylation signal was greatly enhanced in knockout macrophages (Figure 8A).

In the present study, we have shown that tyrosine phosphorylation of TLR2 mediated by SRC and SYK is required for activation and stabilization of TLR2, leading to cytokine production. We observed an enhanced activation of both SRC and SYK following stimulation of WT macrophages by HKSA that was further enhanced in tankyrase-deficient cells (Figure 8B and Supplemental Figure 8A). Additionally, we observed that the interaction between TLR2 and SYK as well as HKSA-mediated tyrosine phosphorylation of TLR2 was enhanced in tankyrase-null macrophages (Figure 8, C and D, and Supplemental Figure 8B). In addition to phosphorylation of TLR2, the half-life of the endogenous TLR2 protein in *Tnks^–/–^Tnks2^fl/fl^ LysM-Cre* macrophages was extended to 42.4 hours compared with that in WT cells (17.5 hours), which resulted in an increase of the TLR2 protein levels (Figure 8, E and F). In view of phosphorylation and stabilization of TLR2, the interaction of TLR2 with MyD88 was enhanced in macrophages lacking endogenous tankyrase compared with WT cells, and this was suppressed by treatment with the inhibitors of SRC and SYK (Figure 8G and Supplemental Figure 8C). Last, we confirmed that high levels of *Tnfa* transcripts observed in macrophages lacking tankyrase were inhibited by the sequential inhibition of SYK and SRC or the combined inhibition of both (Figure 8H). These data conclusively demonstrate that tankyrase is a negative regulator of TLR2 through a phospho-switch in the TIR domain, which controls the ubiquitin-mediated degradation and assembly of a signal transduction complex involving MyD88.

### Endogenous 3BP2 levels in macrophages controlled by tankyrase regulate the innate immune system.

**Figure 9:**
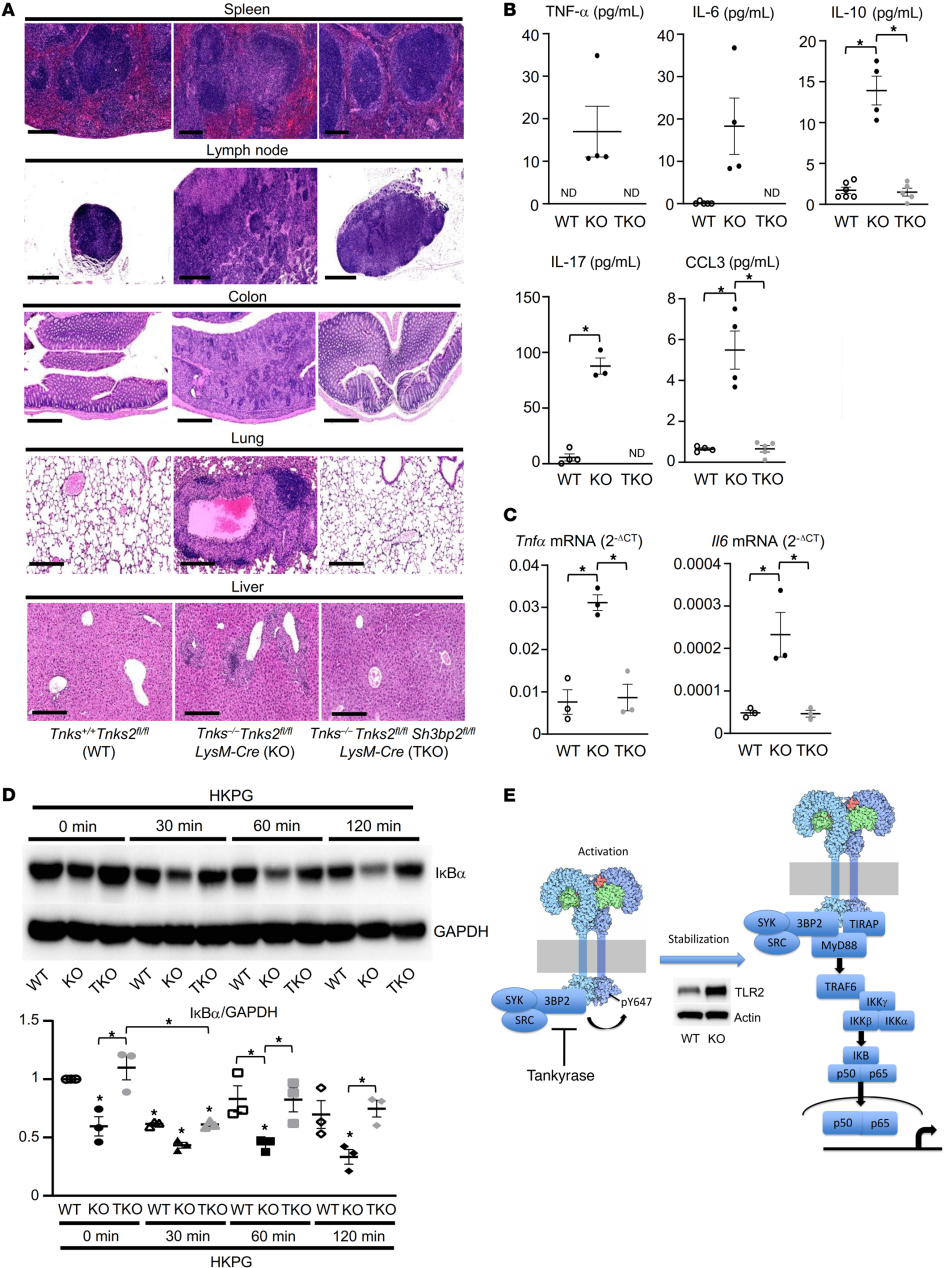
Endogenous 3BP2 levels in macrophages controlled by tankyrase regulate the innate immune system. (**A**) H&E staining of spleen, lymph node, colon, lung, and liver from 12-week-old *Tnks^+/+^* *Tnks2^fl/fl^* (WT), *Tnks^–/–^* *Tnks2^fl/fl^ LysM-Cre* (KO), and *Tnks^–/–^* *Tnks2^fl/fl^ Sh3bp2^fl/fl^ LysM-Cre* (TKO) mice. Scale bars: 250 μm (spleen, lung, and liver), 500 μm (lymph node and colon). (**B**) Serum levels of TNF-α, IL-6, IL-10, IL-17, and CCL3 in 12-week-old *Tnks^+/+^* *Tnks2^fl/fl^* (WT), *Tnks^–/–^* *Tnks2^fl/fl^ LysM-Cre* (KO), and *Tnks^–/–^* *Tnks2^fl/fl^ Sh3bp2^fl/fl^ LysM-Cre* (TKO) mice. ND, not detected. Three dots, 2 dots, and 2 dots are overlapped in the panels of TNF-α, IL-6, and IL-17, respectively. (**C**) qPCR analysis of *Tnfa* and *Il6* mRNA expression in primary murine macrophages derived from *Tnks^+/+^* *Tnks2^fl/fl^* (WT), *Tnks^–/–^* *Tnks2^fl/fl^ LysM-Cre* (KO), and *Tnks^–/–^* *Tnks2^fl/fl^ Sh3bp2^fl/fl^ LysM-Cre* (TKO) mice; *n =* 3. (**D**) Whole-cell lysates from primary murine macrophages derived from *Tnks^+/+^* *Tnks2^fl/fl^* (WT), *Tnks^–/–^* *Tnks2^fl/fl^ LysM-Cre* (KO), and *Tnks^–/–^* *Tnks2^fl/fl^ Sh3bp2^fl/fl^ LysM-Cre* (TKO) mice, starved for 12 hours with 0.1% FBS and cultured in the presence of HKPG (10^7^ cells/mL) for 0–120 minutes, were probed with the indicated antibodies for Western blot analysis. (**E**) Schematic model showing that tankyrase controls innate immunity, cytokine production, and inflammation through degradation of 3BP2, which accelerates SRC- and SYK-mediated TLR2 phosphorylation and activation. The images of TLR2 were adapted with permission from PDB-101, David S. Goodsell and the Research Collaboratory for Structural Bioinformatics (RCSB), Protein Data Bank (PDB) (62). *P* values were determined by ANOVA with Tukey-Kramer post hoc test. Data are presented as mean ± SEM. *P < 0.05.

Last, to determine whether 3BP2 is required for tankyrase-mediated control of TLR2 signaling, we generated *Sh3bp2^fl/fl^* mice intercrossed with the *Tnks^–/–^* *Tnks2^fl/fl^ LysM-Cre* background to generate conditional triple knockout (TKO) mice (*Tnks^–/–^* *Tnks2^fl/fl^ Sh3bp2^fl/fl^ LysM-Cre*) (Supplemental Figure 9A). Consistent with our in vitro data showing that tyrosine phosphorylation of TLR2 by SRC and SYK that are activated by 3BP2 promotes NF-κB–mediated cytokine production, inflammatory bowel disease and the visceral organ histiocytosis observed in *Tnks^–/–^* *Tnks2^fl/fl^ LysM-Cre* mice were abrogated in *Tnks^–/–^* *Tnks2^fl/fl^ Sh3bp2^fl/fl^ LysM-Cre* mice (Figure 9A). Moreover, the accumulation of CD11b- and F4/80-positive cells and elevated cytokines present in the tissues and secondary lymphoid organs of the tankyrase-knockout mice were normalized in the *Tnks^–/–^* *Tnks2^fl/fl^ Sh3bp2^fl/fl^ LysM-Cre* mice (Figure 9B and Supplemental Figure 9B). Activation of NF-κB following HKPG stimulation as evidenced by decreased levels of IκBα protein as well as basal transcript levels of *Tnfa* and *Il6* was restored in macrophages derived from *Tnks^–/–^* *Tnks2^fl/fl^ Sh3bp2^fl/fl^ LysM-Cre* mice (Figure 9, C and D). These data provide strong genetic evidence showing that abundance of endogenous 3BP2 regulated by tankyrase controls innate immunity and inflammation through tyrosine phosphorylation of the TLR-associated proteins including SRC and SYK (Figure 9E).

## Discussion

### Tankyrase regulates the TLR2, 3BP2, SRC, and SYK complex required for innate immunity.

We have previously shown that 3BP2 is negatively regulated by tankyrase through ADP-ribosylation followed by RNF146-mediated polyubiquitylation and proteasomal destruction (15), while missense mutations in the *SH3BP2* gene stabilize 3BP2 proteins, which results in a genetic inflammatory disorder, cherubism (19, 39). We previously reported that 3BP2 and its degradation pathway regulate bone dynamics, lymphocyte development, and energy metabolism. 3BP2 promotes osteoblastogenesis and osteoclastogenesis through activation of ABL and SRC, respectively (40, 41). B and T cell receptor signaling and proliferative expansion are dependent on 3BP2 (42, 43). Mice lacking RNF146 in macrophages suffer from osteoporosis through activation of 3BP2-mediated hyperosteoclastogenesis (44), while deletion of RNF146 in osteoblasts leads to abnormal bone and energy metabolism through osteocalcin (20).

We have uncovered a regulatory mechanism controlling TLR2 signaling by 3BP2 through SRC- and SYK-mediated tyrosine phosphorylation of the TIR domain. We show that 3BP2 forms a signaling module with SRC, SYK, and the TLR2-associated proteins MyD88 and TRAF6, leading to NF-κB activation and cytokine production. Deletion of tankyrase in the myeloid monocytic lineage led to systemic activation of macrophages in the gut, liver, lung, and secondary lymphoid organs with the increased levels of 3BP2 protein. Mice succumb to a lethal autoinflammatory disorder characterized by myeloid monocytic infiltration into visceral organs with high levels of inflammatory cytokines. We show that tankyrase-null macrophages are hyperresponsive to the TLR2 ligands, leading to enhanced NF-κB activation and production of inflammatory cytokines. Deletion of 3BP2 in tankyrase-null myeloid cells rescued the inflammatory phenotype through restoration of the activated NF-κB pathway, demonstrating that tankyrase controls the efficiency of TLR signaling by modulating 3BP2 protein. Notably, the inflammatory phenotype observed in *Tnks^–/–^* *Tnks2^fl/fl^ LysM-Cre* mice is different from that in *Rnf146^fl/fl^ LysM-Cre* mice, which suffer from osteopenia without inflammation (44), demonstrating that tankyrase-mediated ADP-ribosylation of 3BP2 may create a recognition site for other E3 ubiquitin ligases in addition to RNF146.

Tankyrase activates the Wnt/β-catenin pathway through ADP-ribosylation and subsequent degradation of AXIN, a negative regulator of β-catenin (16, 45, 46). Tankyrase-specific inhibitors have been investigated as a potential therapeutic target in β-catenin–dependent cancers (46). Our study suggests that prolonged pharmacologic inhibition of tankyrase could lead to adverse autoinflammatory side effects.

### TLR2 TIR domain Y647 is required for protein stability and TLR2 activity.

Ligand binding to TLR2 results in recruitment of TRAF6 and MyD88 through the TIR domain to induce downstream signaling events, leading to the activation of NF-κB. We show that the signaling module of 3BP2, SRC and SYK constitutes critical proximal components of the TLR2 signaling complex (Figure 9E). We have demonstrated that tyrosines in the TLR2 TIR domain are phosphorylated in an SRC- and SYK-dependent manner. Mutation of all cytoplasmic domain tyrosines abolishes TLR2 signaling, a function that can be fully rescued by the restoration of a single tyrosine at position 647 in the TIR domain, demonstrating the importance of phosphorylation at this site. Tyrosine 647 is required for optimum assembly of TLR2 with 3BP2 and MyD88. Tyrosine 647 in the TIR domain thus functions as a phospho-regulated switch that controls TLR2 activation of NF-κB. Two other tyrosine residues in the cytoplasmic domain of TLR2 have been reported to play a role in signal propagation. Tyrosine 616 is contained in a consensus p85 subunit of the PI3K binding motif, YMKM (47). While mutation of this site alone did not impair binding to PI3K, a double mutation of tyrosine 616 and 761 (YLEW), which has not been identified as a p85 docking site, impaired the association of PI3K, activation of AKT, and recruitment of RAC1 (48). Therefore, the TIR domain function to couple TLR2 to the distinct signaling pathways is required for full activation of NF-κB. Notably, the human TLR2 TIR sequence Y^641^DAFVSY^647^ is conserved in TLR2, TLR4, and TLR7 in humans, mice, rats, and rabbits (Supplemental Figure 9C), suggesting that tyrosine 647 is a conserved phosphotyrosine-dependent regulatory site for other TLR family members. Consistent with this observation, mutations Y674A and Y680A within the YDAFVIY sequence in the TLR4 TIR domain were defective in tyrosine phosphorylation and activation of NF-κB (49, 50). Additionally, we observed that bone marrow–derived *Tnks^–/–^* *Tnks2^fl/fl^ LysM-Cre* macrophages stimulated with not only HKPG (TLR2) but also other TLR ligands, LPS (TLR4) or ssRNA (TLR7), induced elevated IL-6 protein levels in the culture supernatant as well as the *Il6* transcript levels compared with WT cells (Figure 7C, Supplemental Figure 7C, and Supplemental Figure 9, D and E). Therefore, tankyrase may serve to restrain the signaling of other TLR family members in a manner similar to that observed for TLR2. We have thus uncovered a critical phospho-regulated switch in TLR2, regulated by tankyrase, that may be operative in other TLRs.

The observation that a subset of autoantibodies indicative of systemic autoimmunity arises in female mice lacking tankyrase in myeloid cells (Supplemental Figure 7F) suggests that this pathway may play a pathogenic role in the development of human autoimmune diseases in some patients.

Last, an autoinflammatory disorder characterized by inflammation of the bowel in infants similar to the phenotype observed in the *Tnks^–/–^* *Tnks2^fl/fl^ LysM-Cre* mice has recently been described resulting from a gain-of-function mutation in SYK (51). Further studies are warranted to determine whether this phenotype results in part from enhanced phospho-regulated TLR signaling in the gut.

## Methods

### Mice.

The generation of *Tnks^–/–^* and *Sh3bp2^fl/fl^* mice has been described previously (35, 44). *Tnks2^fl/fl^* mice were generated at the Centre for Phenogenomics using the embryonic stem cell clone EPD0282_6_E10 from the Knockout Mouse Project (KOMP) repository at the University of California, Davis (project ID CSD46418). Chimeric mice were generated by diploid aggregation of embryonic stem cells, and the Institute of Cancer Research (ICR) embryos and heterozygous F_1_ mice harboring a targeted *Tnks2* allele were obtained by crossing of chimeric males with C57BL/6 female mice. These mice were crossed with *LysM-Cre* mice expressing Cre recombinase in the myeloid cell lineage including monocytes, macrophages, and granulocytes (The Jackson Laboratory) (52).

### Genotyping of Tnks^–/–^, Tnks2^fl/fl^, and Sh3bp2^fl/fl^ mice.

Tail DNA was extracted using a Wizard SV Genomic DNA Purification System (Promega), and PCR was performed using GoTaq Green Master Mix (Promega) and the following primers: *Tnks* (WT), forward primer 5′-GGATGTGGGCATGCTTAAAA-3′, reverse primer 5′-CACAATGGACCTCAAAGCTG-3′; *Tnks* (KO), forward primer 5′-ACGTAAACTCCTCTTCAGACCTAATAAC-3′, reverse primer 5′-CACAATGGACCTCAAAGCTG-3′; *Tnks2*, forward primer 5′-CAGTGTCGTCTTCAGCTTGG-3′, reverse primer 5′-TGTACACATGAGCTCTGGGT-3′; *Sh3bp2*, forward primer 5′-ACAGCTCAGTGTTGGATTCCTGGCTC-3′, reverse primer 5′-ACCCTGACTGGTCGGTGTTTCAGAAC-3′.

### Cell culture.

All cultures were maintained in a 5% CO_2_ environment at 37°C. Bone marrow cells were cultured in α-MEM (Gibco, Thermo Fisher Scientific) supplemented with 10% FBS (Wisent) and 2% CMG (conditioned medium supernatant containing recombinant M-CSF) (53). HEK293T cells (ATCC) were cultured in DMEM supplemented with 10% FBS.

### Immunofluorescence.

Primary murine macrophages grown on glass coverslips were fixed with 4% formaldehyde for 10 minutes, permeabilized, and incubated with the indicated antibody. Following incubation with secondary antibody, nuclei were stained with DAPI (Life Technologies, Thermo Fisher Scientific). Slides were mounted using Aqua Mount (Thermo Fisher Scientific). Confocal imaging was performed with an Olympus IX81 inverted microscope and FluoView software (Olympus).

### Cytokine analysis.

Mouse cytokines and chemokines in the serum were analyzed using the Mouse Magnetic Luminex Screening Assay (R&D Systems) or the Mouse IL-6 ELISA Kit (MilliporeSigma or RayBiotech).

### Reagents and antibodies.

Unless stated otherwise, all chemicals were purchased from MilliporeSigma. Antibodies were obtained from the following sources: anti–p-Tyr (catalog 9411), anti–p-SRC (Y416) (catalog 2101), anti-SRC (catalog 2109), anti–p-SYK (Y525/526) (catalog 2710), anti-SYK (catalog 2712), anti–p-IκBα (catalog 2859), anti-IκBα (catalog 9242), anti-GAPDH (catalog 2118), anti-TLR2 (catalog 12276 for human and 13744 for mouse), anti–NF-κB (catalog 3033), and anti–NF-κB (catalog 8242) from Cell Signaling Technology; anti-MyD88 (catalog SAB3500472) and anti-FLAG M2 (catalog F3165) from MilliporeSigma; anti-Myc (catalog sc-40) and anti-actin (catalog sc-47778) from Santa Cruz Biotechnology; anti-HA (M180-3) from MBL International; anti–p-Tyr (4G10) (catalog 05-321) from Millipore; anti-PPP1R11 (ab171960) from Abcam; and anti-3BP2 (catalog H00006452-M01) from Abnova. Halt Protease and Phosphatase Inhibitor Cocktail was from Thermo Fisher Scientific. SYK inhibitor was from Millipore. PP2 and BMS 345541 were from Selleckchem.

### RNA extraction and quantitative real-time PCR analysis.

Total cellular RNA was extracted using the RNeasy Plus Mini Kit (QIAGEN). The ImProm-II Reverse Transcription System (Promega) was used for reverse transcription, and quantitative real-time PCR was performed on the Step One Plus Real-Time PCR System (Applied Biosystems). The sequences of primers were as follows: *Rpl19*, forward primer 5′-CTGAAGGTCAAAGGGAATGTG-3′, reverse primer 5′-GGACAGAGTCTTGATGATCTC-3′; *Tnfa*, forward primer 5′-TTGACCTCAGCGCTGAGTTG-3′, reverse primer 5′-CCTGTAGCCCACGTCGTAGC-3′; *Il6*, forward primer 5′-TGATGCACTTGCAGAAAACA-3′, reverse primer 5′-ACCAGAGGAAATTTTCAATAGGC-3′; and *Tnks*, forward primer 5′-CCCACTTCATAATGCCTGCT-3′, reverse primer 5′-GCTTTTGCTGAAGGATCTGC-3′. The relative expression of each mRNA normalized by the expression of *Rpl19* was calculated by the ΔCt method.

### Western blot analysis and coimmunoprecipitation.

Cells were lysed with Nonidet P-40 (NP-40) buffer (20 mM Tris [pH 8.0], 137 mM NaCl, 1% NP-40, 2 mM EDTA) or RIPA buffer (50 mM Tris [pH 7.5], 150 mM NaCl, 1% NP-40, 0.1% SDS, 0.25% sodium deoxycholate, 1 mM EDTA) supplemented with protease and phosphatase inhibitors as described previously (41). Lysates were cleared by centrifugation for 10 minutes at 20,000*g* and 4°C. Immunoprecipitation was performed at 4°C with the indicated antibodies, and the products were collected on Dynabeads Protein A or G (Life Technologies, Thermo Fisher Scientific). GST-tagged protein was purified with Glutathione Sepharose 4B (GE Healthcare). For Western blotting, protein in whole-cell lysates was resolved by SDS-PAGE and transferred to PVDF membranes (Immobilon; MilliporeSigma). Membranes were blocked in 5% BSA or 5% nonfat dried milk in PBST (PBS plus 0.1% Tween-20). The images are representative of 3 independent experiments. The relative integrated densities of each protein band were digitized by ImageJ (NIH) and are shown in the figures.

### Histology.

Tissues were fixed in 10% neutral-buffered formalin and embedded in paraffin. Sections were stained with H&E. IHC was performed using anti-F4/80 (Abcam, ab6640), anti-Mac2 (Cedarlane, CL8942AP), anti–CSF-1R (Abcam, ab215441), anti-CD45R (BD Biosciences, 550286), anti-MPO (Abcam, ab9535), and anti-EPX (Biorbyt, orb5168) antibodies, and the antigen-antibody binding was detected using Simple Stain MAX PO (Nichirei) and the DAB Substrate Kit (Nichirei).

### Mass spectrometry.

In order to immunoprecipitate TLR2 protein complexes, HEK293T cells were lysed in NP-40 buffer (1% NP-40, 0.5% deoxycholate, 50 mM Tris [pH 7.5], 150 mM NaCl, 10% glycerol, 25 mM NaF, 1 mM EDTA, and protease and phosphatase inhibitors) and subjected to immunoaffinity purification using immobilized anti-Myc affinity agarose beads as described previously (54, 55). For affinity purification–mass spectrometry (AP-MS), isolated immune complexes were eluted with 0.15% trifluoroacetic acid and processed for downstream proteomic analysis (54, 55). For MS sample preparation, tryptic peptides were concentrated and purified on homemade C18 columns or C18 StageTips (Thermo Fisher Scientific) before liquid chromatography–tandem MS. Peptides were separated by reverse-phase chromatography using a nanoflow ultra-performance liquid chromatography (UPLC) system (Thermo Fisher Scientific) with a linear gradient. UPLC was coupled online to an Orbitrap Elite or Q-Exactive or Lumos mass spectrometer (Thermo Fisher Scientific). Peptide ions were fragmented by collision-induced dissociation. For AP-MS samples, raw MS files acquired from the MS analyzer were processed using MaxQuant software (version 1.3.0.5) according to the standard workflow (56). MS/MS spectra were searched against the UniProt human proteome (release 2017) using the Andromeda search engine (57). For statistical evaluation of data, a false discovery rate of 0.01 was set for peptide and protein identification. Protein label-free quantification (LFQ) intensity was chosen as the quantitative value representing protein abundance and used for calculations of protein differential expression. Protein LFQ intensities across different samples were first normalized by the intensities of the TLR2 baits. Raw MS files acquired from the MS analyzer were also processed by Scaffold software (Proteome Software). SRC peptides were identified from a TLR2 AP-MS sample by MS/MS shown by peptide tandem mass spectra. The sequences of the peptides derived from TLR2 spectra are shown highlighted in yellow in whole protein sequences.

### In vivo ubiquitin assay.

In vivo ubiquitin assay was performed as described previously (41, 58). Briefly, HEK293T cells were transiently cotransfected with expression vectors of HA-tagged ubiquitin (HA-Ub) and the indicated constructs. Twenty-four hours after transfection, cells were treated with 10 mM MG132 for 4 hours. Then cells were lysed in complete cell lysis buffer (2% SDS, 150 mM NaCl, 10 mM Tris-HCl [pH 8.0]) with protease and phosphatase inhibitors, boiled for 10 minutes, sheared with a sonication device, and incubated at 4°C for 30 minutes with dilution buffer (10 mM Tris-HCl [pH 8.0], 150 mM NaCl, 2 mM EDTA, 1% Triton). After lysates were cleared by centrifugation for 30 minutes at 14,000*g* and 4°C, immunoprecipitation and Western blot were performed with the indicated antibodies.

### Transient transfection and luciferase assay.

To assess the NF-κB transcriptional activity, we used the NF-κB–luciferase reporter construct (NF-κB-luc; Promega). HEK293T cells were transiently cotransfected with NF-κB-luc reporter plasmid and phRL-CMV plasmid (Promega) with or without the indicated constructs using the LipoD293 DNA In Vitro Transfection Reagent (SignaGen Laboratories). Cells were lysed and assayed for firefly and Renilla luciferase activity using the Dual-Glo Luciferase Assay System (Promega). The data were expressed as the ratio of firefly to Renilla activity.

### Nonradioactive pulse-chase assay.

Newly synthesized protein was labeled with Click-IT Metabolic Labeling Reagents for Proteins (Invitrogen, Thermo Fisher Scientific) according to the manufacturer’s instructions as described previously (59). Primary murine macrophages or HEK293T cells cotransfected with the indicated constructs were seeded and cultured in methionine-free medium for an hour. Cells were then cultured in methionine-free medium containing 50 μM of AHA (a methionine analog; Invitrogen) for 4 hours. After washing with PBS, the medium was changed with complete medium, and the cells were chased for the indicated time. The cells were lysed in the lysis buffer (1% SDS, 50 mM Tris-HCl [pH 8.0], and protease inhibitor), and AHA-incorporated protein was biotinylated using the Click-IT Protein Reaction Buffer Kit (Invitrogen, Thermo Fisher Scientific) and Biotin Alkyne (Invitrogen, Thermo Fisher Scientific). After precipitation and dissolving, biotinylated protein was collected with Dynabeads M-280 Streptavidin (Invitrogen, Thermo Fisher Scientific). The purified proteins were probed with the indicated antibodies for Western blot analysis.

### Plasmids.

Myc-TLR2 (WT) plasmid was generated by amplifying human TLR2 (with a Myc tag added to the C-terminus; included in the oligonucleotide) and cloning it into the XhoI site of pEF-BOS. Myc-TLR2 (6YF) plasmid was generated by amplifying gBlock fragments (Integrated DNA Technologies) and cloning them into the XhoI site of pEF-BOS (Myc tag added to the C-terminus; included in the oligonucleotide). Myc-TLR2 (add-back) plasmids were generated by overlap extension PCR using primers with the desired mutations and cloning into the XhoI site of pEF-BOS. GST-MyD88 plasmid was generated by amplifying mouse *Myd88* (MGC clone) and cloning it into the BamHI/NotI sites of pEBG. GST-PPP1R11 plasmid was generated by amplifying human PPP1R11 from pCMV6 XL5 human PPP1R11 (OriGene) and cloning it in frame into the NotI/BamHI sites of pEBG. Flag-3BP2 plasmid (p3Xflag-CMV-10) was generated by amplifying mouse *Sh3bp2* and cloning it into the EcoRI site of p3Xflag-CMV-10. HA-TRAF6 plasmid was generated by amplifying mouse *Traf6* (MGC clone) and cloning it into the BamHI site of pSGT (HA tag on N-terminus; included in oligonucleotide).

### Flow cytometric analysis.

Single-cell suspensions of the colon, lung, spleen, lymph node, and peripheral blood from mice were stained with a combination of fluorescently labeled antibodies on ice. All samples were analyzed by the LSRFortessa X-20 or FACSAria III Cell Sorter (BD Biosciences) and FlowJo software.

### Antigen microarrays.

Antigen microarrays were generated using 153 antigens as described previously (38). Briefly, antigens were spotted in duplicate onto FAST nitrocellulose slides (GVS) using a Virtek microarrayer. The arrays were blocked overnight in blocking buffer (5% FCS in PBS with 0.1% Tween-20). The arrays were then probed with mouse serum diluted 1:100 with blocking buffer at 4°C for 1 hour. After washing, the arrays were probed with Cy3-labeled goat anti-mouse IgG Fc and Cy5-labeled goat anti–mouse IgM secondary antibodies (Jackson ImmunoResearch Laboratories) diluted in blocking buffer at 4°C for 45 minutes. The arrays were scanned on an Axon 4200A scanner (Molecular Devices), and median fluorescence intensities were quantified using Genepix Version 6.1 (Molecular Devices). Significance analysis of microarrays was used to determine significant changes in antigen reactivity between WT and knockout mice with a false discovery rate of 5% (*q* values less than 0.05) (60).

### Statistics.

All results are shown as means ± SEM of data from at least 3 separate experiments. The data were subjected to ANOVA with Tukey-Kramer post hoc test or unpaired, 2-tailed *t* test with JMP 7 (SAS Institute) to determine differences. *P* values of less than 0.05 were accepted as statistically significant.

### Study approval.

All animal studies were approved by the Animal Research Council at University Health Network, Toronto, Canada, and by the IRB of the Okayama University Graduate School of Medicine, Dentistry and Pharmaceutical Sciences, Okayama, Japan.

## Author contributions

YM and RR designed the experiments. YM performed experiments and analyzed the results. IDD, JLR, ML, SC, NL, HAA, JT, MFM, AC, FH, YA, TK, KS, and JW performed specific experiments and analyzed the results. YM and RR wrote the manuscript with helpful revision by HAA.

## Supplementary Material

Supplemental data

Supplemental table 1

## Version 1 04/01/2022

Electronic publication
