# Supplementary material for: Tankyrase represses autoinflammation through the attenuation of TLR2 signaling
Source: J Clin Invest. 2022 Apr 1;132(7):e140869. doi: 10.1172/JCI140869 (PMC8970677; doi:10.1172/JCI140869)
Supplement: Supplemental data [file jci-132-140869-s065.pdf]

Supplemental Information (Titles and Legends for Supplemental Figures)

Supplemental Figure 1. TIR domain tyrosines are required for optimal TLR2 signaling. (A) Primary murine macrophages derived from wild-type mice were starved for 12 hours with 0.1% FBS and cultured in the presence or absence of HKSA (10^7 cells/ml) for 20 minutes, and MyD88 immune complexes were probed with the indicated antibodies for western blot analysis. (B-D) HEK293T cells were co-transfected with the indicated constructs, and Flag-3BP2 immune complexes were probed with the indicated antibodies for western blot analysis. (E) Flow cytometric analysis of TLR2 expression on the cell surface of HEK293T cells co-transfected with or without TLR2 (WT). n = 3. Representative flow cytometry plots (left) and the frequency (right, mean ± SEM (%)) of TLR2-expressing cells are presented. (F) Three SRC-unique peptides, highlighted in yellow, were identified from a TLR2 AP-MS sample by MS/MS. (G) Luciferase activity from a NF-κB reporter assay in HEK293T cells co-transfected with the indicated constructs and cultured in a serum-free medium in the presence or absence of PP2 (10 μM). n = 3. (H-J) HEK293T cells were co-transfected with the indicated constructs, and Myc-TLR2 (H), HA-SYK (I) or Flag-TLR2 (J) immune complexes as well as the input and whole cell lysates in Figure 1, J (H), K (I) or L (J) were probed with the indicated antibodies for western blot analysis. P values were determined by unpaired t-test (A and E) or ANOVA with Tukey–Kramer's post hoc test (G). Data are presented as mean ± SEM. *$P < 0.05$.

Supplemental Figure 2. TIR domain tyrosines are required for the stability of TLR2. (A) Cycloheximide-chase to ascertain TLR2 protein stability. HEK293T cells expressing TLR2 (WT) or TLR2 (6YF) were treated with cycloheximide for the indicated time intervals and lysed, and TLR2 protein levels were determined by western blot analysis. The percentages of TLR2 protein levels were plotted as a function of time. n = 3. (B) HEK293T cells were co-transfected with the indicated constructs, and Myc-TLR2 immune complexes were probed with the indicated antibodies for western blot analysis. (C) HEK293T cells co-transfected with the indicated constructs and cultured in the presence or absence of SYK inhibitor (10 μM) and PP2 (10 μM) were lysed, and GST-MyD88 immune complexes and whole cell lysates (W.C.L) were probed with the indicated antibodies for western blot analysis. P values were determined by unpaired t-test. Data are presented as mean ± SEM. *$P < 0.05$.

Supplemental Figure 3. TLR2 tyrosine 647 is a phospho-switch that regulates TLR2 stabilization and activation. (A-C) Whole cell lysates in Figure 3B (A), 3F (B) and 3G (C) were probed with the indicated antibodies for western blot analysis.

Supplemental Figure 4. Mice lacking Tankyrase in myeloid cells exhibit systemic inflammation. (A) Schematic diagrams of the $Tnks2^{loxP/loxP}$ allele. (B) Genotyping PCR of $Tnks^{+/+}Tnks2^{fl/fl}$ and $Tnks^{-/-}Tnks2^{fl/fl}$ *LysM-Cre* mice using primers shown in the Methods section. For *Tnks*, the wild-type (WT) product is 412 bp, and the knockout (KO) product is 274 bp. For *Tnks2*, the wild-type product is 258 bp, and the floxed product is 388 bp. The Cre

product is 480 bp. (C) qPCR analysis of *Tnks* and *Tnks2* mRNA expression in primary murine macrophages derived from *Tnks*$^{+/+}$*Tnks2*$^{fl/fl}$ (WT) and *Tnks*$^{-/-}$*Tnks2*$^{fl/fl}$ *LysM-Cre* (KO) mice. n = 4. (D-F) H&E staining of the stomach (D), ileum (E) and cecum (F) from 12-week-old *Tnks*$^{+/+}$*Tnks2*$^{fl/fl}$ and *Tnks*$^{-/-}$*Tnks2*$^{fl/fl}$ *LysM-Cre* mice. Scale bar: 250 µm. P values were determined by unpaired t-test. Data are presented as mean ± SEM. *P < 0.05.

Supplemental Figure 5. Tankyrase regulates inflammation by controlling innate and adaptive immunity. (A and B) MPO (A) and EPX (B) immunostaining of spleen, lymph node, the colon, lung and liver from 12-week-old *Tnks*$^{+/+}$*Tnks2*$^{fl/fl}$ (WT) and *Tnks*$^{-/-}$*Tnks2*$^{fl/fl}$ *LysM-Cre* (KO) mice. Scale bar: 100 µm.

Supplemental Figure 6. Tankyrase regulates inflammation by controlling innate and adaptive immunity. (A-E) Flow cytometric analysis of CD11b, F4/80, CD11c, B220, CD4 and CD8 expression on the cell surface and IL-6 expression in CD4 positive cells of spleen (A), lymph node (B), the colon (C), lung (D) and peripheral blood (E) from 12-week-old *Tnks*$^{+/+}$*Tnks2*$^{fl/fl}$ (WT) and *Tnks*$^{-/-}$*Tnks2*$^{fl/fl}$ *LysM-Cre* (KO) mice. n = 3. Representative flow cytometry plots of cells (far left), the absolute cell numbers (middle) and the mean fluorescence intensity (MFI) of IL-6 (far right) are presented as mean ± SEM. P values were determined by unpaired t-test. *P < 0.05. (F and G) The representative gating strategy (F) and histogram (G) of IL-17- or IL-6-expressing CD4 positive T lymphocytes of spleen, lymph node, the colon, lung and peripheral blood from 12-week-old *Tnks*$^{+/+}$*Tnks2*$^{fl/fl}$ (WT, red line) and *Tnks*$^{-/-}$*Tnks2*$^{fl/fl}$ *LysM-Cre* (KO, blue line) mice.

Supplemental Figure 7. Tankyrase restrains TLR signaling and the production of inflammatory cytokines. (A) Serum levels of IL-2, IL-4, IL-5, IL-9, IL-12, CCL2, CCL11 and CXCL1 in 12-week-old *Tnks*$^{+/+}$*Tnks2*$^{fl/fl}$ (WT) and *Tnks*$^{-/-}$*Tnks2*$^{fl/fl}$ *LysM-Cre* (KO) mice. N.D.: not detected. (B) The IL-6 protein levels in the culture supernatant of bone marrow-derived macrophages starved for 12 hours in 0.1% FBS and cultured in medium for 24 hours (left) or cell lysates of macrophages (right) from *Tnks*$^{+/+}$*Tnks2*$^{fl/fl}$ (WT) and *Tnks*$^{-/-}$*Tnks2*$^{fl/fl}$ *LysM-Cre* (KO) mice were measured by ELISA. n = 3. (C) qPCR analysis of *Il6* mRNA expression in primary murine macrophages derived from *Tnks*$^{+/+}$*Tnks2*$^{fl/fl}$ (WT) and *Tnks*$^{-/-}$*Tnks2*$^{fl/fl}$ *LysM-Cre* (KO) mice, starved for 12 hours with 0.1% FBS and cultured in the presence of HKPG (10^7 cells/ml) for 0-24 hours. n = 3. (D) Relative integrated densities of each protein band in Figure 7E were quantified with statistical analysis. (E) Whole cell lysates from primary murine macrophages derived from *Tnks*$^{+/+}$*Tnks2*$^{fl/fl}$ (WT) and *Tnks*$^{-/-}$*Tnks2*$^{fl/fl}$ *LysM-Cre* (KO) mice, starved for 12 hours with 0.1% FBS and cultured in the presence of HKPG (10^7 cells/ml) for 0-120 minutes were probed with the indicated antibodies for western blot analysis. (F) Autoantibodies in the serum from *Tnks*$^{+/+}$*Tnks2*$^{fl/fl}$ (WT) and *Tnks*$^{-/-}$*Tnks2*$^{fl/fl}$ *LysM-Cre* (KO) mice were detected by antigen microarrays as described in the Methods section and shown as a cluster heatmap. Yellow indicates high mean fluorescence intensity (MFI) whereas blue indicates low MFI as shown in the scale. P values were determined by unpaired t-test (A and B) or ANOVA with Tukey–Kramer's post hoc test (C-E). Data are presented as mean ± SEM. *P < 0.05.

Supplemental Figure 8. Tyrosine phosphorylation of TLR2 regulates NF-κB-mediated cytokine production. (A and B) Whole cell lysates in Figure 8A (A) and 8D (B) were probed with the indicated antibodies for western blot analysis. (C) Primary murine macrophages derived from $Tnks^{-/-}Tnks2^{fl/fl}$ *LysM-Cre* mice, starved for 12 hours with 0.1% FBS, cultured in the presence or absence of SYK inhibitor (10 μM) and PP2 (10 μM) for 3 hours and stimulated with HKSA (10^7 cells/ml) for 20 minutes were lysed, and MyD88 immune complexes were probed with the indicated antibodies for western blot analysis. P values were determined by unpaired t-test (C) or ANOVA with Tukey–Kramer's post hoc test (A and B). Data are presented as mean ± SEM. *P < 0.05.

Supplemental Figure 9. Endogenous 3BP2 levels in macrophages controlled by Tankyrase regulates the innate immune system. (A) Whole cell lysates from primary murine macrophages derived from $Tnks^{+/+}Tnks2^{fl/fl}$ (WT), $Tnks^{-/-}Tnks2^{fl/fl}$ *LysM-Cre* (KO) and $Tnks^{-/-}Tnks2^{fl/fl}$ $Sh3bp2^{fl/fl}$ *LysM-Cre* (TKO) mice were probed with the indicated antibodies for western blot analysis. (B) FACS analysis of CD11b and F4/80 expression on the cell surface of spleen, lymph node, the colon, lung and peripheral blood from 12-week-old $Tnks^{+/+}Tnks2^{fl/fl}$ (WT), $Tnks^{-/-}Tnks2^{fl/fl}$ *LysM-Cre* (KO) and $Tnks^{-/-}Tnks2^{fl/fl}$ $Sh3bp2^{fl/fl}$ *LysM-Cre* (TKO) mice. n = 3. (C) The human TLR2 TIR sequence from Y641 to Y647 "YDAFVSY" is conserved in TLR2, 4, and 7 in all the species. (D) qPCR analysis of *Il6* mRNA expression in primary murine macrophages derived from $Tnks^{+/+}Tnks2^{fl/fl}$ (WT) and $Tnks^{-/-}Tnks2^{fl/fl}$ *LysM-Cre* (KO) mice, starved for 12 hours with 0.1% FBS and cultured in the presence LPS (100 ng/ml) or ssRNA (0.5 μg/ml) for 0-24 hours. n = 3. (E) Primary murine macrophages derived from $Tnks^{+/+}Tnks2^{fl/fl}$ (WT) and $Tnks^{-/-}Tnks2^{fl/fl}$ *LysM-Cre* (KO) mice were starved for 12 hours with 0.1% FBS and cultured in the presence or absence of LPS (100 ng/ml) or ssRNA (0.5 μg/ml) for 24 hours. The IL-6 protein levels in the culture supernatant were measured by ELISA. n = 3. P values were determined by ANOVA with Tukey–Kramer's post hoc test. Data are presented as mean ± SEM. *P < 0.05.

Supplemental Table 1. Affinity Purification Mass Spectrometric Analysis of TLR2. A list of 54 proteins identified from a mass spectrometry of TLR2 shown in Figure 1D and Supplemental Figure 1F.

Supplemental Figure 1

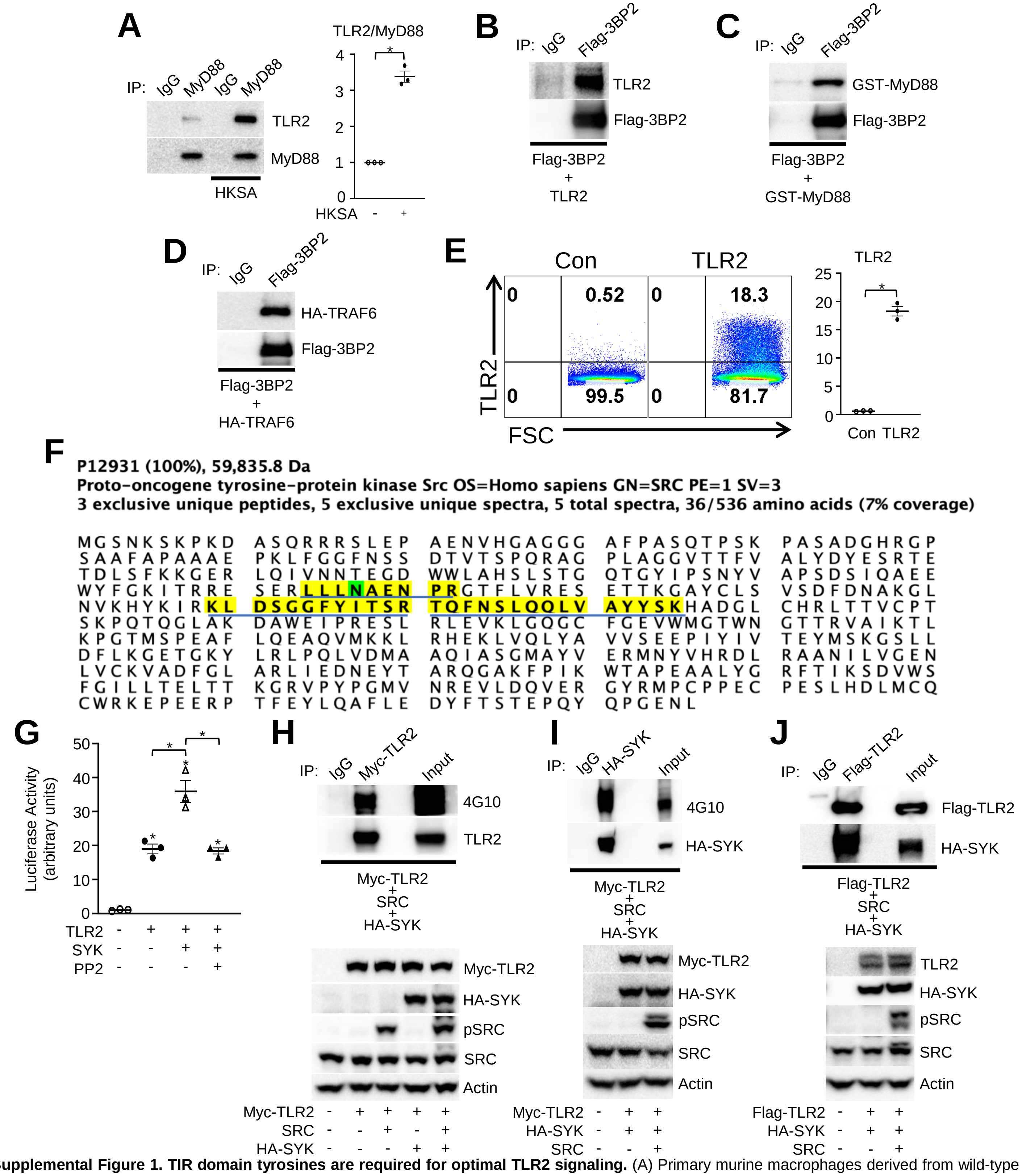

Supplemental Figure 1. TIR domain tyrosines are required for optimal TLR2 signaling. (A) Primary murine macrophages derived from wild-type mice were starved for 12 hours with 0.1% FBS and cultured in the presence or absence of HKSA (10^7 cells/ml) for 20 minutes, and MyD88 immune complexes were probed with the indicated antibodies for western blot analysis. (B-D) HEK293T cells were co-transfected with the indicated constructs, and Flag-3BP2 immune complexes were probed with the indicated antibodies for western blot analysis. (E) Flow cytometric analysis of TLR2 expression on the cell surface of HEK293T cells co-transfected with or without TLR2 (WT). n = 3. Representative flow cytometry plots (left) and the frequency (right, mean ± SEM (%)) of TLR2-expressing cells are presented. (F) Three SRC-unique peptides, highlighted in yellow, were identified from a TLR2 AP-MS sample by MS/MS. (G) Luciferase activity from a NF-κB reporter assay in HEK293T cells co-transfected with the indicated constructs and cultured in a serum-free medium in the presence or absence of PP2 (10 μM). n = 3. (H-J) HEK293T cells were co-transfected with the indicated constructs, and Myc-TLR2 (H), HA-SYK (I) or Flag-TLR2 (J) immune complexes as well as the input and whole cell lysates in Figure 1, J (H), K (I) or L (J) were probed with the indicated antibodies for western blot analysis. P values were determined by unpaired t-test (A and E) or ANOVA with Tukey–Kramer's post hoc test (G). Data are presented as mean ± SEM. *P < 0.05.

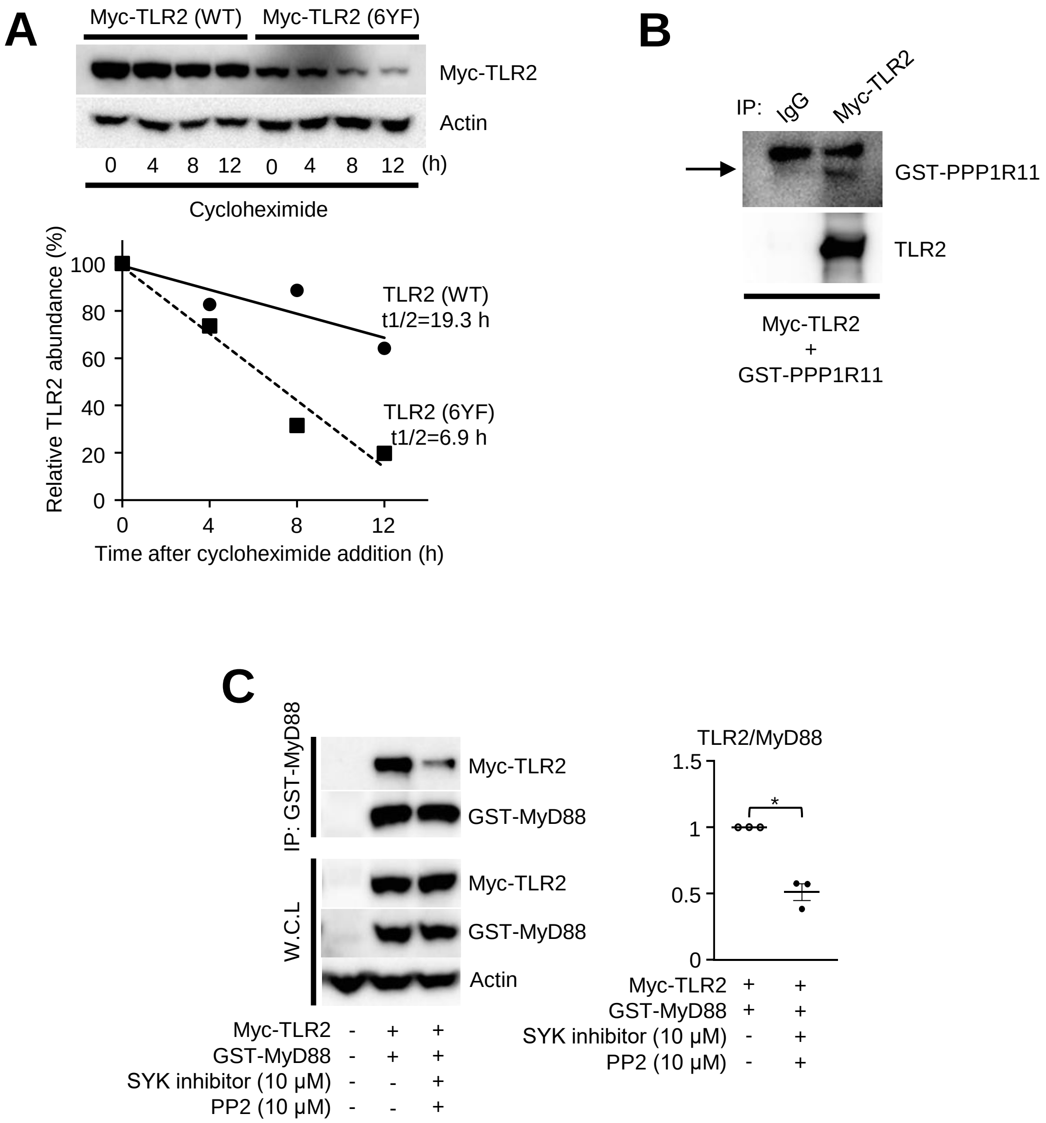

Supplemental Figure 2. TIR domain tyrosines are required for the stability of TLR2. (A) Cycloheximide-chase to ascertain TLR2 protein stability. HEK293T cells expressing TLR2 (WT) or TLR2 (6YF) were treated with cycloheximide for the indicated time intervals and lysed, and TLR2 protein levels were determined by western blot analysis. The percentages of TLR2 protein levels were plotted as a function of time. n = 3. (B) HEK293T cells were co-transfected with the indicated constructs, and Myc-TLR2 immune complexes were probed with the indicated antibodies for western blot analysis. (C) HEK293T cells co-transfected with the indicated constructs and cultured in the presence or absence of SYK inhibitor (10 μM) and PP2 (10 μM) were lysed, and GST-MyD88 immune complexes and whole cell lysates (W.C.L) were probed with the indicated antibodies for western blot analysis. P values were determined by unpaired t-test. Data are presented as mean ± SEM. *$P < 0.05$.

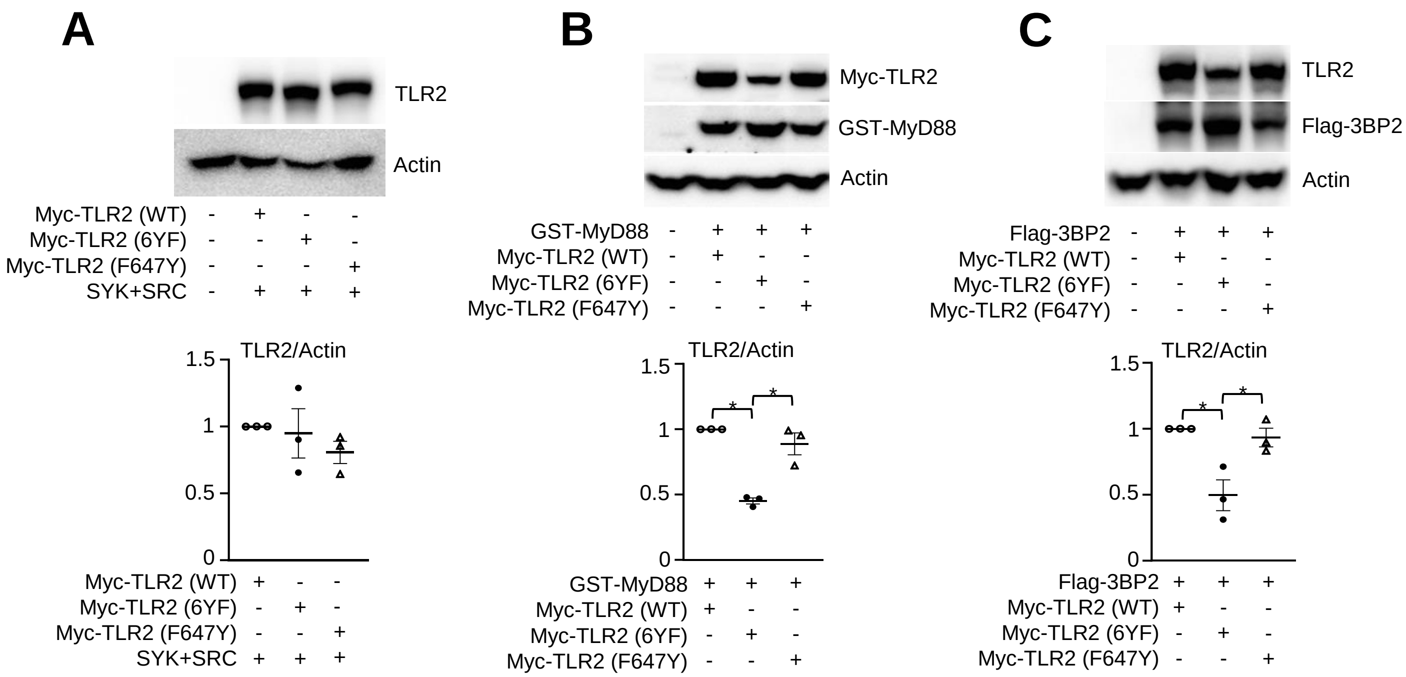

Supplemental Figure 3. TLR2 tyrosine 647 is a phospho-switch that regulates TLR2 stabilization and activation. (A-C) Whole cell lysates in Figure 3B (A), 3F (B) and 3G (C) were probed with the indicated antibodies for western blot analysis.

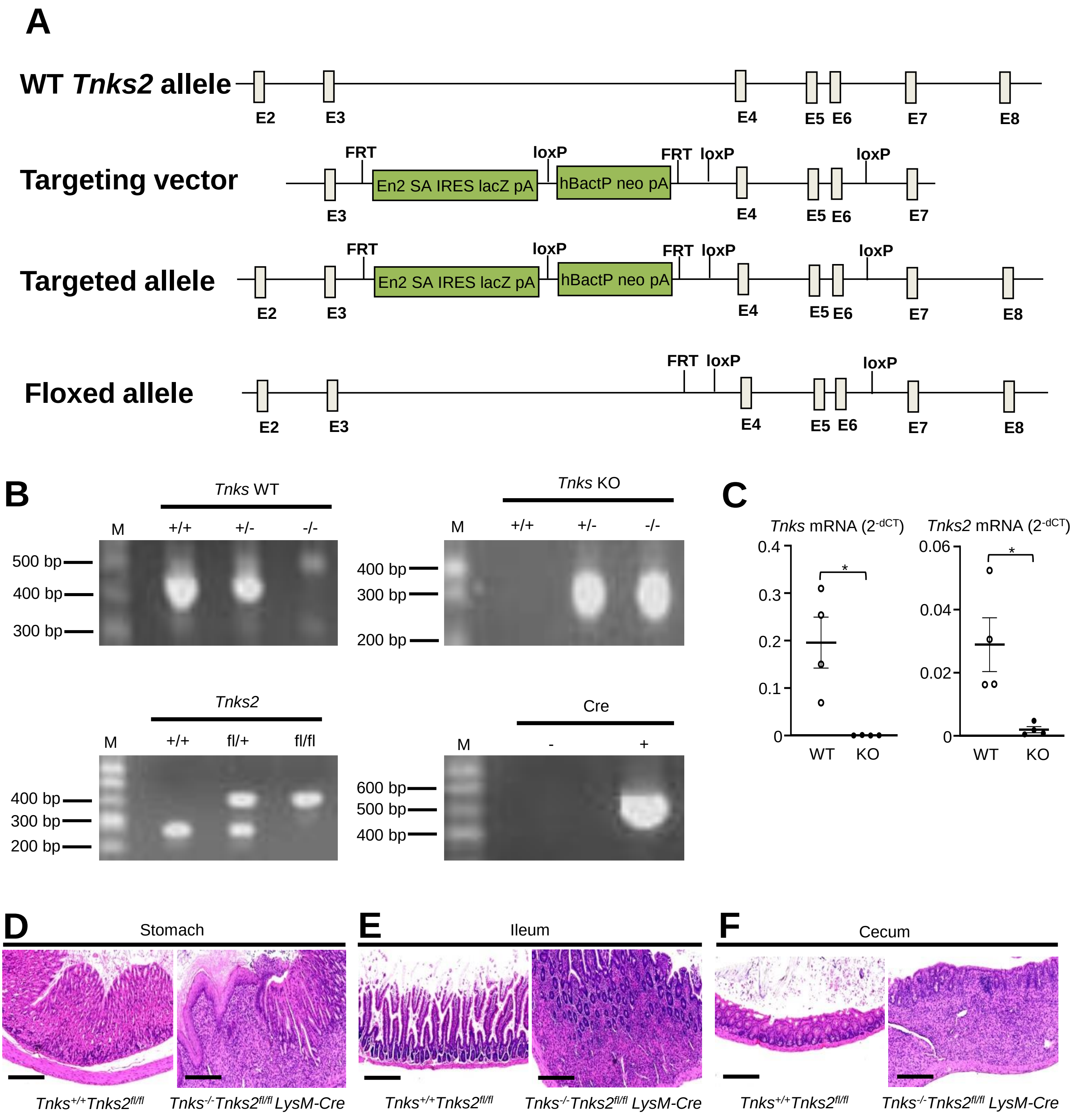

Supplemental Figure 4. Mice lacking Tankyrase in myeloid cells exhibit systemic inflammation. (A) Schematic diagrams of the *Tnks2*$^{loxP/loxP}$ allele. (B) Genotyping PCR of *Tnks*$^{+/+}$*Tnks2*$^{fl/fl}$ and *Tnks*$^{-/-}$*Tnks2*$^{fl/fl}$ *LysM-Cre* mice using primers shown in the Methods section. For *Tnks*, the wild-type (WT) product is 412 bp, and the knockout (KO) product is 274 bp. For *Tnks2*, the WT product is 258 bp, and the floxed product is 388 bp. The Cre product is 480 bp. (C) qPCR analysis of *Tnks* and *Tnks2* mRNA expression in primary murine macrophages derived from *Tnks*$^{+/+}$*Tnks2*$^{fl/fl}$ (WT) and *Tnks*$^{-/-}$*Tnks2*$^{fl/fl}$ *LysM-Cre* (KO) mice. n = 4. (D-F) H&E staining of the stomach (D), ileum (E) and cecum (F) from 12-week-old *Tnks*$^{+/+}$*Tnks2*$^{fl/fl}$ and *Tnks*$^{-/-}$*Tnks2*$^{fl/fl}$ *LysM-Cre* mice. Scale bar: 250 μm. P values were determined by unpaired t-test. Data are presented as mean ± SEM. *P < 0.05.

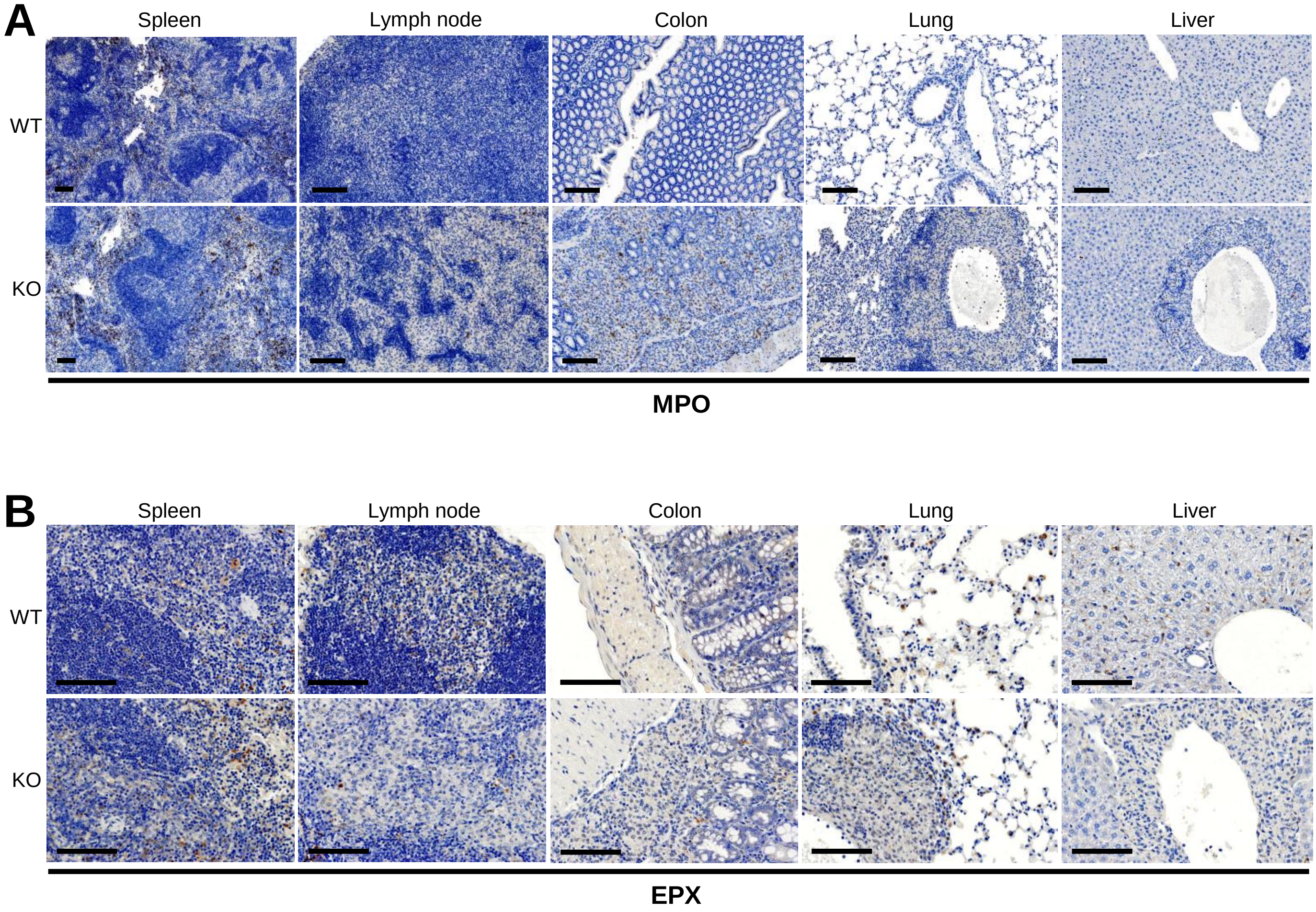

Supplemental Figure 5. Tankyrase regulates inflammation by controlling innate and adaptive immunity. (A and B) MPO (A) and EPX (B) immunostaining of spleen, lymph node, the colon, lung and liver from 12-week-old *Tnks*$^{+/+}$*Tnks2*$^{fl/fl}$ (WT) and *Tnks*$^{-/-}$ *Tnks2*$^{fl/fl}$ *LysM-Cre* (KO) mice. Scale bar: 100 μm.

Supplemental Figure 6

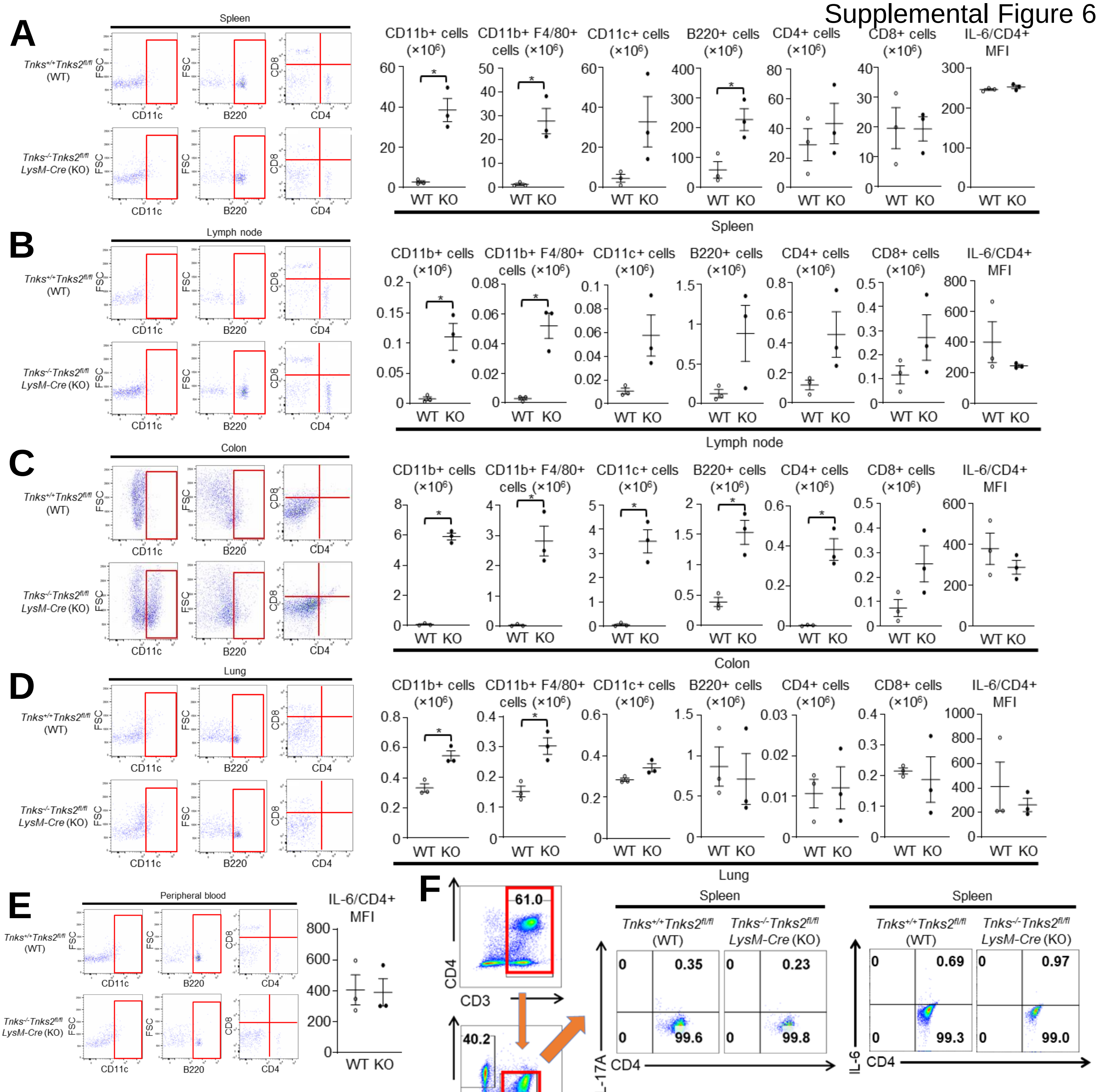

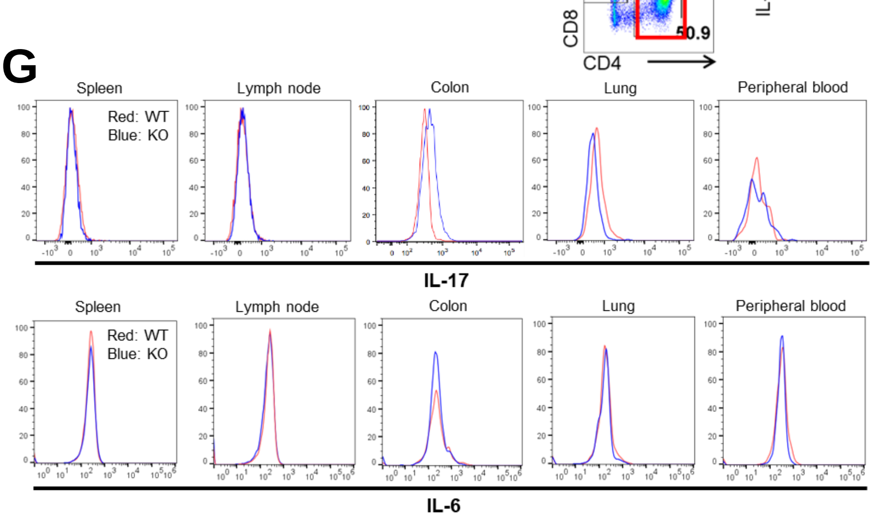

Supplemental Figure 6. Tankyrase regulates inflammation by controlling innate and adaptive immunity. (A-E) Flow cytometric analysis of CD11b, F4/80, CD11c, B220, CD4 and CD8 expression on the cell surface and IL-6 expression in CD4 positive cells of spleen (A), lymph node (B), the colon (C), lung (D) and peripheral blood (E) from 12-week-old *Tnks$^{+/+}$Tnks2$^{fl/fl}$* (WT) and *Tnks$^{-/-}$Tnks2$^{fl/fl}$ LysM-Cre* (KO) mice. n = 3. Representative flow cytometry plots of cells (far left), the absolute cell numbers (middle) and the mean fluorescence intensity (MFI) of IL-6 (far right) are presented as mean ± SEM. P values were determined by unpaired t-test. *$P < 0.05$. (F and G) The representative gating strategy (F) and histogram (G) of IL-17- or IL-6-expressing CD4 positive T lymphocytes of spleen, lymph node, the colon, lung and peripheral blood from 12-week-old *Tnks$^{+/+}$Tnks2$^{fl/fl}$* (WT, red line) and *Tnks$^{-/-}$Tnks2$^{fl/fl}$ LysM-Cre* (KO, blue line) mice.

Supplemental Figure 7

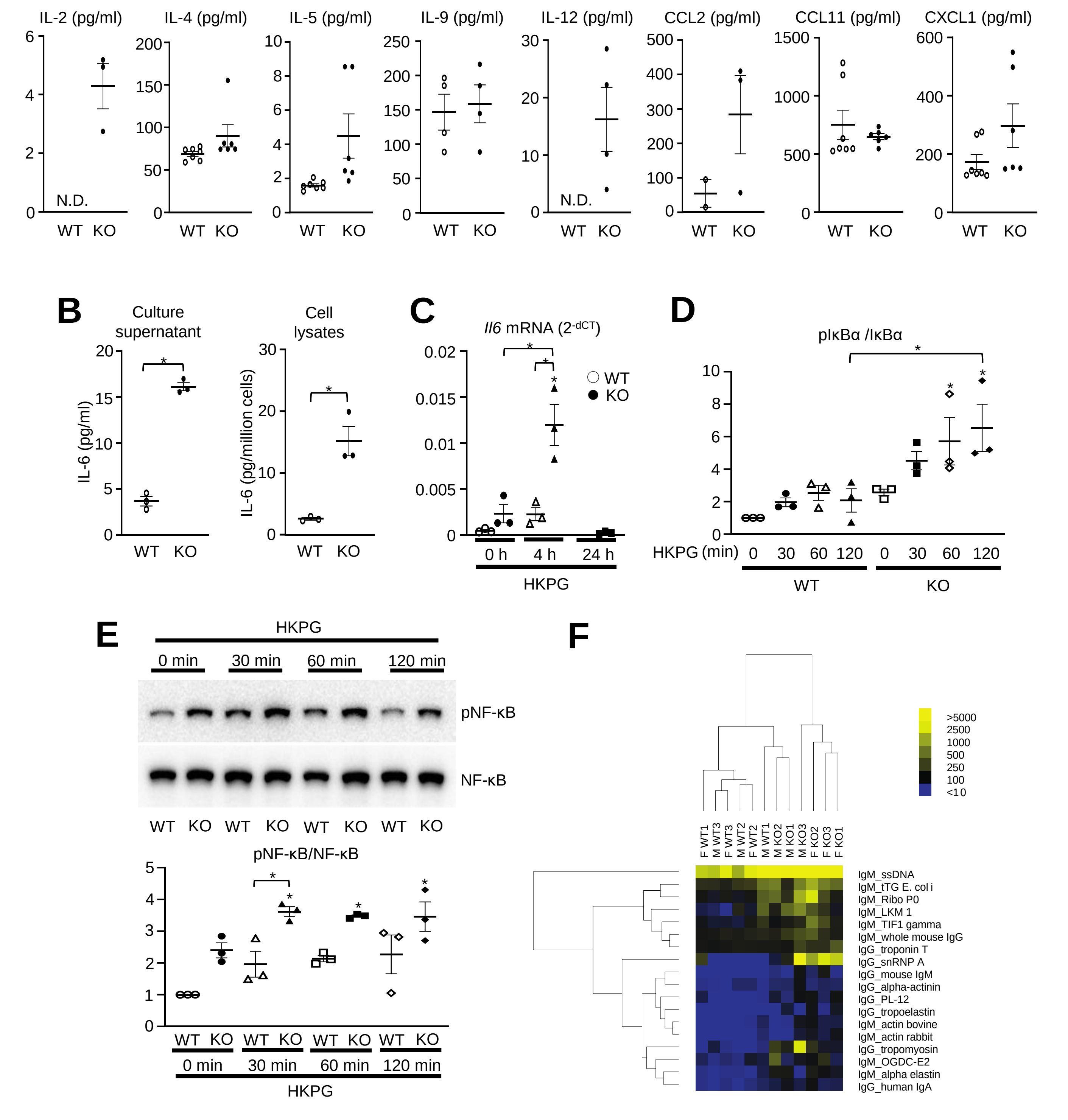

Supplemental Figure 7. Tankyrase restrains TLR signaling and the production of inflammatory cytokines. (A) Serum levels of IL-2, IL-4, IL-5, IL-9, IL-12, CCL2, CCL11 and CXCL1 in 12-week-old *Tnks*$^{+/+}$*Tnks2*$^{fl/fl}$ (WT) and *Tnks*$^{-/-}$*Tnks2*$^{fl/fl}$ *LysM-Cre* (KO) mice. N.D.: not detected. (B) The IL-6 protein levels in the culture supernatant of bone marrow-derived macrophages starved for 12 hours in 0.1% FBS and cultured in medium for 24 hours (left) or cell lysates of macrophages (right) from *Tnks*$^{+/+}$*Tnks2*$^{fl/fl}$ (WT) and *Tnks*$^{-/-}$*Tnks2*$^{fl/fl}$ *LysM-Cre* (KO) mice were measured by ELISA. n = 3. (C) qPCR analysis of *Il6* mRNA expression in primary murine macrophages derived from *Tnks*$^{+/+}$*Tnks2*$^{fl/fl}$ (WT) and *Tnks*$^{-/-}$*Tnks2*$^{fl/fl}$ *LysM-Cre* (KO) mice, starved for 12 hours with 0.1% FBS and cultured in the presence of HKPG (10^7 cells/ml) for 0-24 hours. n = 3. (D) Relative integrated densities of each protein band in Figure 7E were quantified with statistical analysis. (E) Whole cell lysates from primary murine macrophages derived from *Tnks*$^{+/+}$*Tnks2*$^{fl/fl}$ (WT) and *Tnks*$^{-/-}$*Tnks2*$^{fl/fl}$ *LysM-Cre* (KO) mice, starved for 12 hours with 0.1% FBS and cultured in the presence of HKPG (10^7 cells/ml) for 0-120 minutes were probed with the indicated antibodies for western blot analysis. (F) Autoantibodies in the serum from *Tnks*$^{+/+}$*Tnks2*$^{fl/fl}$ (WT) and *Tnks*$^{-/-}$*Tnks2*$^{fl/fl}$ *LysM-Cre* (KO) mice were detected by antigen microarrays as described in the Methods section and shown as a cluster heatmap. Yellow indicates high mean fluorescence intensity (MFI) whereas blue indicates low MFI as shown in the scale. P values were determined by unpaired t-test (A and B) or ANOVA with Tukey–Kramer's post hoc test (C-E). Data are presented as mean ± SEM. *P < 0.05.

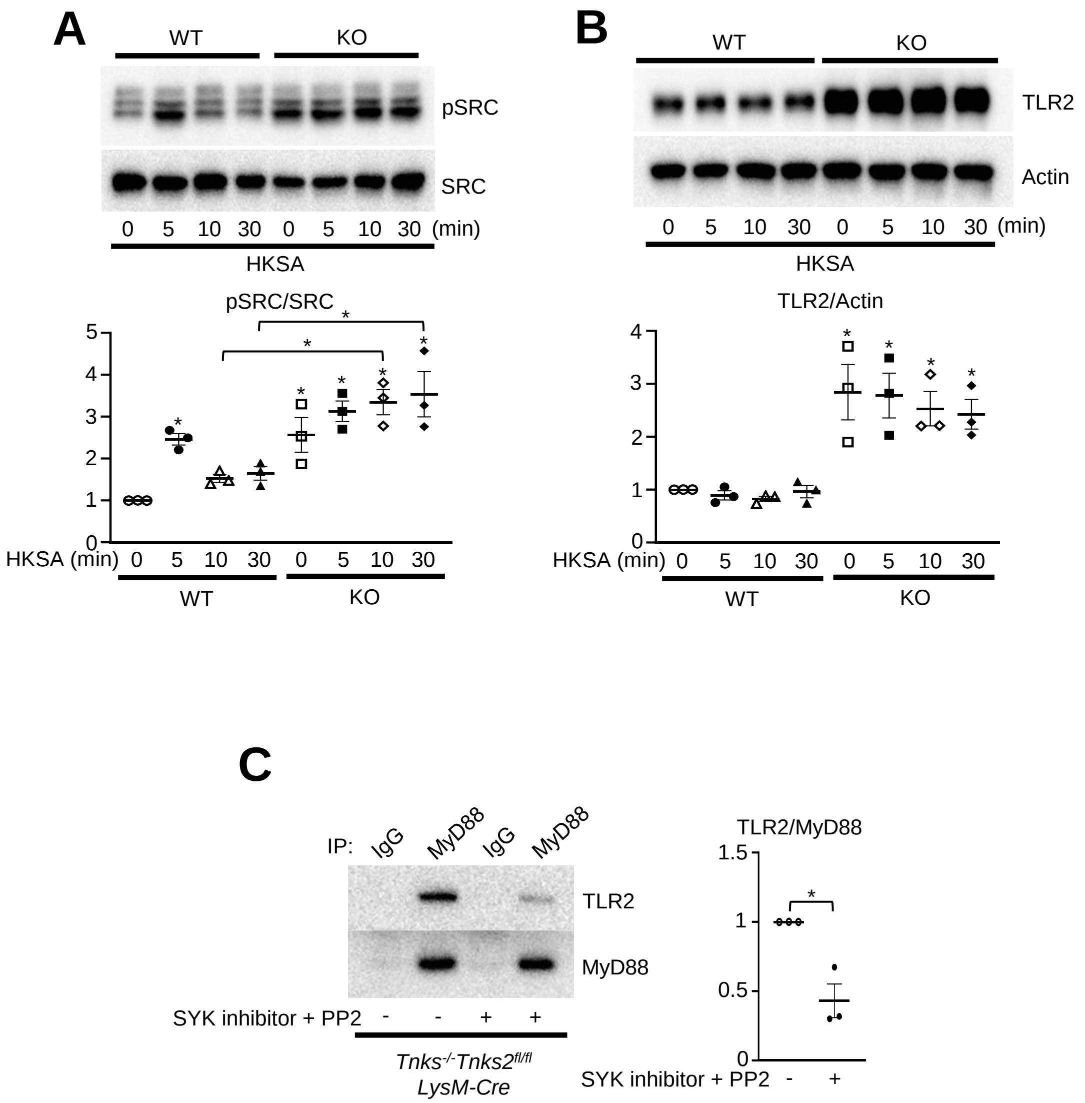

Supplemental Figure 8. Tyrosine phosphorylation of TLR2 regulates NF-κB-mediated cytokine production. (A and B) Whole cell lysates in Figure 8A (A) and 8D (B) were probed with the indicated antibodies for western blot analysis. (C) Primary murine macrophages derived from *Tnks*$^{-/-}$*Tnks2*$^{fl/fl}$ *LysM-Cre* mice, starved for 12 hours with 0.1% FBS, cultured in the presence or absence of SYK inhibitor (10 μM) and PP2 (10 μM) for 3 hours and stimulated with HKSA (10^7 cells/ml) for 20 minutes were lysed, and MyD88 immune complexes were probed with the indicated antibodies for western blot analysis. P values were determined by unpaired t-test (C) or ANOVA with Tukey–Kramer's post hoc test (A and B). Data are presented as mean ± SEM. *P < 0.05.

Supplemental Figure 9

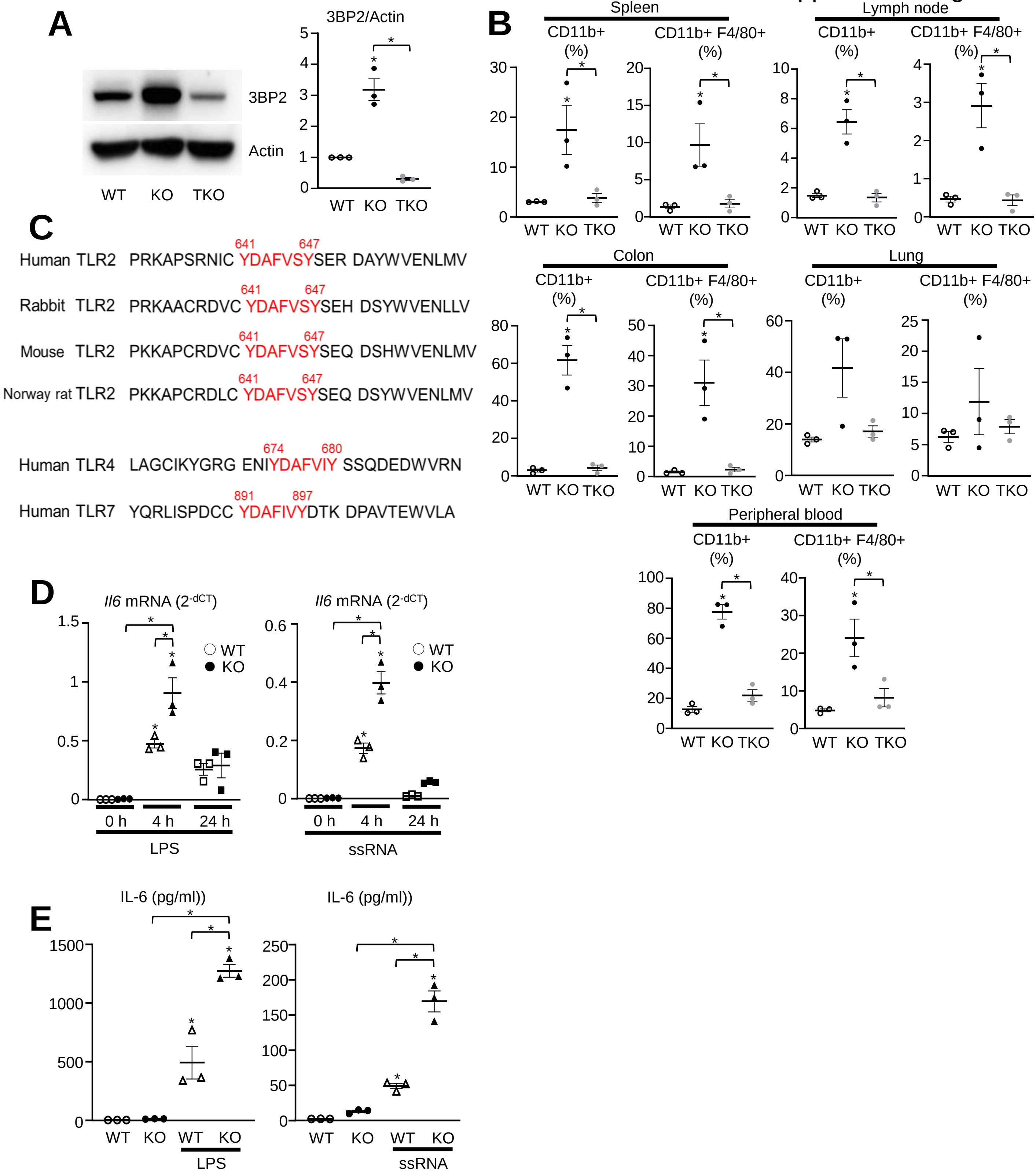

Supplemental Figure 9. Endogenous 3BP2 levels in macrophages controlled by Tankyrase regulates the innate immune system. (A) Whole cell lysates from primary murine macrophages derived from *Tnks*$^{+/+}$*Tnks2*$^{fl/fl}$ (WT), *Tnks*$^{-/-}$*Tnks2*$^{fl/fl}$ *LysM-Cre* (KO) and *Tnks*$^{-/-}$*Tnks2*$^{fl/fl}$ *Sh3bp2*$^{fl/fl}$ *LysM-Cre* (TKO) mice were probed with the indicated antibodies for western blot analysis. (B) FACS analysis of CD11b and F4/80 expression on the cell surface of spleen, lymph node, the colon, lung and peripheral blood from 12-week-old *Tnks*$^{+/+}$*Tnks2*$^{fl/fl}$ (WT), *Tnks*$^{-/-}$*Tnks2*$^{fl/fl}$ *LysM-Cre* (KO) and *Tnks*$^{-/-}$*Tnks2*$^{fl/fl}$ *Sh3bp2*$^{fl/fl}$ *LysM-Cre* (TKO) mice. n = 3. (C) The human TLR2 TIR sequence from Y641 to Y647 "YDAFVSY" is conserved in TLR2, 4, and 7 in all the species. (D) qPCR analysis of *Il6* mRNA expression in primary murine macrophages derived from *Tnks*$^{+/+}$*Tnks2*$^{fl/fl}$ (WT) and *Tnks*$^{-/-}$*Tnks2*$^{fl/fl}$ *LysM-Cre* (KO) mice, starved for 12 hours with 0.1% FBS and cultured in the presence LPS (100 ng/ml) or ssRNA (0.5 µg/ml) for 0-24 hours. n = 3. (E) Primary murine macrophages derived from *Tnks*$^{+/+}$*Tnks2*$^{fl/fl}$ (WT) and *Tnks*$^{-/-}$*Tnks2*$^{fl/fl}$ *LysM-Cre* (KO) mice were starved for 12 hours with 0.1% FBS and cultured in the presence or absence of LPS (100 ng/ml) or ssRNA (0.5 µg/ml) for 24 hours. The IL-6 protein levels in the culture supernatant were measured by ELISA. n = 3. P values were determined by ANOVA with Tukey–Kramer's post hoc test. Data are presented as mean ± SEM. *P < 0.05.
